# Record linkage studies of drug-related deaths among adults who were released from prison to the community: a scoping review

**DOI:** 10.1186/s12889-023-15673-0

**Published:** 2023-05-05

**Authors:** Janine A. Cooper, Ifeoma Onyeka, Christopher Cardwell, Euan Paterson, Richard Kirk, Dermot O’Reilly, Michael Donnelly

**Affiliations:** 1 Centre for Public Health, Queen’s University Belfast, Royal Hospitals Site, Grosvenor Road, Belfast, UK; 2 Administrative Data Research Centre Northern Ireland (ADRC NI), Centre for Public Health, Queen’s University Belfast, Royal Hospitals Site, Grosvenor Road, Belfast, UK; 3 Present address: Department of Psychology, Sociology and Politics, Sheffield Hallam University, Sheffield, UK; 4 Healthcare in Prison, South Eastern Health and Social Care Trust, Dundonald, UK

**Keywords:** Record linkage, Data linkage, Drug-related deaths, Mortality, Prison, Former prisoners, Scoping review

## Abstract

**Background:**

There are public health concerns about an increased risk of mortality after release from prison. The objectives of this scoping review were to investigate, map and summarise evidence from record linkage studies about drug-related deaths among former adult prisoners.

**Methods:**

MEDLINE, EMBASE, PsychINFO and Web of Science were searched for studies (January 2011- September 2021) using keywords/index headings. Two authors independently screened all titles and abstracts using inclusion and exclusion criteria and subsequently screened full publications. Discrepancies were discussed with a third author. One author extracted data from all included publications using a data charting form. A second author independently extracted data from approximately one-third of the publications. Data were entered into Microsoft Excel sheets and cleaned for analysis. Standardised mortality ratios (SMRs) were pooled (where possible) using a random-effects DerSimonian-Laird model in STATA.

**Results:**

A total of 3680 publications were screened by title and abstract, and 109 publications were fully screened; 45 publications were included. The pooled drug-related SMR was 27.07 (95%CI 13.32- 55.02; I 2 = 93.99%) for the first two weeks (4 studies), 10.17 (95%CI 3.74–27.66; I 2 = 83.83%) for the first 3–4 weeks (3 studies) and 15.58 (95%CI 7.05–34.40; I 2 = 97.99%) for the first 1 year after release (3 studies) and 6.99 (95%CI 4.13–11.83; I 2 = 99.14%) for any time after release (5 studies). However, the estimates varied markedly between studies. There was considerable heterogeneity in terms of study design, study size, location, methodology and findings. Only four studies reported the use of a quality assessment checklist/technique.

**Conclusions:**

This scoping review found an increased risk of drug-related death after release from prison, particularly during the first two weeks after release, though drug-related mortality risk remained elevated for the first year among former prisoners. Evidence synthesis was limited as only a small number of studies were suitable for pooled analyses for SMRs due to inconsistencies in study design and methodology.

**Supplementary Information:**

The online version contains supplementary material available at 10.1186/s12889-023-15673-0.

## Background

The world prison population size was over 10.7 million in 2021 or 140 per 100,000 of population [1]. However, the prison population is estimated to be more than 11.5 million when we take into account statistical information about prisoners which is unavailable or unrecognised internationally or is missing from published national prison population sizes [1]. Prison population rates vary by country and region. For example, the USA has the highest prison population—over 2 million people, equivalent to a rate of 629 per 100,000 [1]. There are higher rates of mental and physical health problems in prison populations compared to the general population, and substance use disorders are common in people who are committed to prison [2, 3]. There is a risk of disruption to treatment and care and a deterioration in health when former prisoners transition from prison to living in the community [2]. Furthermore, negative health effects may be compounded by post-release experiences of former prisoners including loss of social support, enduring stigma, financial insecurity and difficulties obtaining stable housing [4].

There are concerns about the increased risk of mortality after release from prison and the contribution of drug-related causes to deaths in former prisoners [5–7]. A review in 2010 reported that 76% of deaths in the first 2 weeks after release and 59% of deaths within the first 3 months of release were due to drug-related causes [7]. There is a need to examine the range of potential factors that may contribute to the increased risk of drug-related deaths after release from prison, including decreased tolerance following relative abstinence in prison and the concurrent use of multiple drugs [8]. Observational studies investigating the risk of mortality after prison release often use large administrative datasets to link prison and death records. An updated review of the evidence in this area, including the extent of the literature, methodologies, findings and gaps in knowledge is warranted and a scoping review approach has been chosen to map key concepts and summarise evidence in this field. This scoping review updates and maps research evidence in the area of record linkage studies of drug-related deaths among former adult prisoners, and identifies and profiles at-risk former prisoners. The findings are discussed in terms of their contribution to potential interventions and to informing future research and policy. This review was undertaken as part of a work programme in the Administrative Data Research Centre, Northern Ireland and in response to concerns from public health, criminal justice, voluntary and community groups and wider society about prisoner health and well-being in Northern Ireland after release from prison.

## Methods

We chose to conduct a scoping review because of the broader scope of our review that included a focus on how the research was conducted and differences in methodologies used among record-linkage studies in this research area. This broader scope was informed largely by the results of previous systematic reviews/meta-analysis regarding reported high levels of heterogeneity [5–7]. The methods used to conduct this scoping review have been published previously as a protocol [9] and a summary of the methodology is provided here. This review followed the first five stages of the framework for conducting scoping reviews by Arksey and O’Malley [10] and adhered to the guidance developed by the Joanna Briggs Institute (JBI) and the JBI Collaboration. For example, as recommended by the JBI, the population, concept and context (PCC) guide was incorporated into the scoping review title, research questions and inclusion criteria [11]. In addition, the Preferred Reporting Items for Systematic reviews and Meta-Analyses extension for Scoping Reviews (PRISMA-ScR) checklist and guidance was used to structure and report this review [12]. This scoping review was structured to meet the requirements of the PRISMA-ScR checklist. A completed PRISMA-ScR checklist (used to report this work) has been provided as supplementary material in this scoping review.

### Stage 1: identifying the research question

The following questions were addressed by the scoping review:

- 1. What is the scope of the literature on record linkage studies of drug-related deaths among former adult prisoners who have been released to the community?
- 2. How is research conducted on this topic?
- 3. What methodologies are used?
- 4. What are the findings in relation to mortality?
- 5. Where are the knowledge gaps on this topic?

### Stage 2: identifying relevant studies

In order to summarise the most recent evidence, the start date of 2011 was chosen for this scoping review. Four bibliographic databases (MEDLINE, EMBASE, PsychINFO and Web of Science) were searched for studies from January 2011 to September 2021 using keywords and index headings (modified as required for each database). The search terms related to ‘mortality’, ‘drugs’ and ‘ex-prisoner’ (and their variants). The review focused on drug-related deaths and as such, the search strategy included a broad range of terms including substance-related disorders, drug overdose and drug misuse. The full list is found in appendices 1, 2, 3, and 4. The search strategy for MEDLINE was developed by JAC and MD with assistance from the Subject Librarian for the School of Medicine, Dentistry and Biomedical Sciences in Queen’s University Belfast, and was published with the review protocol [9]. JAC and MD developed search strategies for EMBASE, PsychINFO and Web of Science, and all search strategies used in this review have been provided as supplementary material (appendices 1, 2, 3, and 4). The reference lists of included studies were screened by JAC to identify any additional publications. Due to the absence of resources for translation, all search strategies were limited to publications in the English language. There was no geographical restrictions on studies.

### Stage 3: study selection

All bibliographic database searches were performed by JAC on 15^th^ September 2021 and the results were combined in Endnote Reference Manager where duplicate publications were subsequently removed. JAC and IO independently screened all titles and abstracts using the pre-defined inclusion criteria and excluded any non-eligible publications. Publications were screened using criteria defined in Table 1. Publications were screened in full if an abstract was not available and/or there was uncertainty over inclusion. Subsequently, JAC and IO independently screened full publications for inclusion and any discrepancies between JAC and IO regarding eligibility were resolved in a discussion between JAC and MD during which a unanimous decision was made. No authors of publications were contacted during this process.

**Table 1:** Modifications made to inclusion and exclusion criteria as part of review

**Inclusion and exclusion criteria defined in protocol **[9]	**Modifications made to inclusion and exclusion criteria as part of review**
**Population**	
The population will include adults (defined as 18 years and older) who have been imprisoned and released to the community. Individuals released from custodial placements such as young offender institutions will be excluded. Individuals remaining in prison custody (eg, prisoners on remand and sentenced prisoners) will be excluded. There will be no exclusion on gender	During the screening of publications, it became apparent that the age definition for inclusion into the adult prison population differed in various regions. We therefore modified this criterion to include any definition of adult prison population. No other changes were made
**Concept**	
The key concepts revolve around record linkage of drug-related deaths in adults who have been imprisoned. Included studies must use data linkage (or similar meaning terms) to determine mortality outcomes following release from prison. Studies with no data linkage will be excluded. Only studies reporting cause-specific mortality (ie, drug-related deaths) for either the entire study population or a subset of the study population will be included	No changes were made
**Context**	
All geographical locations will be included. The review will include research from peer-review journals. Qualitative studies, commentaries, editorials and conference abstracts will be excluded	No changes were made

### Stage 4: charting the data

A draft charting form was piloted as part of the protocol development stage. As part of the review process, the charting form was retested by JAC and EP (the final data charting form used is provided in appendix 5). JAC independently extracted information from all included publications using this data charting form. The accuracy and consistency of the recorded information was checked by using a second reviewer (EP) to independently extract information for a proportion of the included publications (*n* = 14) and resolving any discrepancies via discussion by team members. In addition, JAC and MD met weekly and discussed the studies in the review particularly for their fit with the pre-specified inclusion criteria and the charting procedure.

### Stage 5: collating, summarising and reporting the results

Information was extracted from the charting forms and entered into Microsoft Excel sheets for data management and analysis. Data in the Microsoft Excel sheets were subsequently cleaned and extracted information was summarised. The data were analysed and presented in a format that was designed to answer the scoping review questions and organised according to the main conceptual categories including methodology, key findings and gaps in the research. All descriptive tables and figures for this review were prepared using these data contained in the Microsoft Excel sheets. We reported mortality outcomes following release from prison in terms of, for example, crude mortality rates (CMRs) and standardised mortality ratios (SMRs). Where possible, age, sex/gender and race/ethnicity, time period examined after prison release and information on specific drugs were reported in relation to drug-related mortality. SMRs for drug-related deaths after release from incarceration were pooled statistically, where possible. The log SMR was determined as well as the Standard Error (SE) of the log SMR from the published SMR and confidence intervals (CIs). In meta-analysis, the consistency of effects across studies should be assessed [13]. The random-effects DerSimonian-Laird model was used. In STATA version 16.1 [StataCorp, College Station, Texas, USA], the *meta* command was used to compute effect sizes and summarise data and produce forest plots. The heterogeneity was measured using the I^2^ squared statistic and testing using a formal chi-squared test for heterogeneity. Meta-analyses are not a usual feature of the methodology of scoping reviews [10, 11], however, exploratory meta-analyses were conducted following this scoping review to deepen the level of critical analysis by, for example, assessing in a quantitative way, the consistency of effects. Meta-analyses were performed posteriori and were not planned in the study protocol [9].

## Patient and public involvement

Our empirical study of prisoner post-release mortality and this scoping review were initiated in response to concerns about the increasing number of drug-related deaths generally from the UK Chief Medical Officers (CMOs) including the CMO for Northern Ireland. We continue to consult with, and involve, key prison health care staff including the Clinical Director of Healthcare in Prisons in Northern Ireland in our ongoing programme of prison health research (co-author of this paper).

## Results

### Study selection

**Fig. 1:**
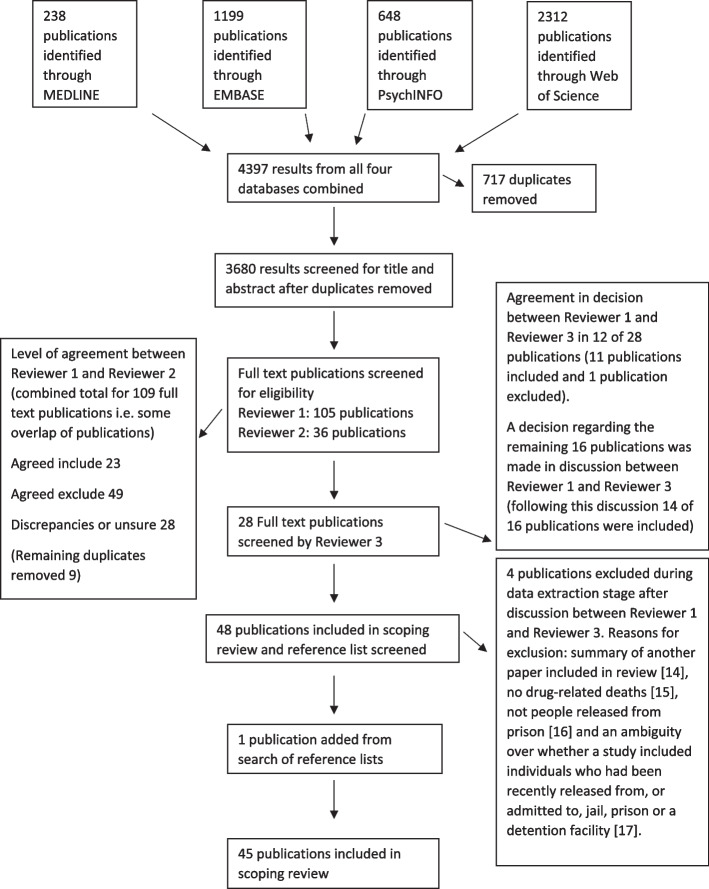
Flow diagram showing the number of publications at each stage of the review process

The search strategy identified a combined total of 4397 publications across four bibliographic databases. Using the Endnote duplicate tool, 717 duplicate publications were removed. A total of 3680 publications were screened by title and abstract; 109 publications were deemed to meet eligibility criteria and full-text publications were screened by two authors (reviewer 1 fully screened 105 publications and reviewer 2 fully screened 36 publications i.e. there was some overlap of screened publications). Authors noted that some remaining duplicate articles were among the publications excluded at this stage (9 remaining duplicates removed). There was agreement between reviewers to include 23 publications and exclude 49 publications. There was disagreement or uncertainty between reviewers about 28 publications and these publications were fully screened by reviewer 3 and resolved via discussion with reviewer 1; 25 of these 28 publications were included. A total of 48 publications were included at this stage and the reference lists of included publications were screened, resulting in the addition of one further publication. Four publications were excluded during the data extraction stage after discussion between reviewer 1 and reviewer 3. The reasons for exclusion of publications were as follows: summary of another paper included in review [14], no drug-related deaths [15], not people released from prison [16] and an ambiguity over whether a study included individuals who had been recently released from, or admitted to, jail, prison or a detention facility [17]. Following this review process, a total of 45 publications were included. A flow diagram for each stage is presented in Fig. 1.

Study characteristics and methods for included studies are shown in Tables 2 and 3, respectively.

### Research questions

The data were analysed in a format that was designed to answer the review questions, as presented below.

- 1. What is the scope of the literature on record linkage studies of drug-related deaths among former adult prisoners who have been released to the community?
  The included studies (*n* = 45) were published across 25 different journals. The five most common journals that published studies in this area were Addiction (*n* = 12), Drug and Alcohol Dependence (*n* = 7), American Journal of Public Health (*n* = 2), JAMA Psychiatry (*n* = 2) and Public Health Reports (*n* = 2) (Table 2). The remaining included studies (*n* = 20) were published in 20 different journals. The geographical distribution of the included studies, by location of the custody setting, shows a total of 9 countries/regions (appendix 6). The most common locations were the USA (*n* = 24) and Australia (*n* = 7). Other locations were Canada, Denmark, Norway, Sweden, Taiwan and the UK. One publication included both USA and Australia by way of comparing cohorts [18]. The search strategy included January 2011 to September 2021 in order to summarise the most recent evidence (the distribution of publications across this time period is presented in appendix 6).
  This scoping review focused on studies of mortality risk during the time period after release from incarceration—the number of years and months following release from incarceration were provided in 60% of studies (*n* = 27) (Table 3). Information about incarceration dates was provided in half of those studies without release dates (*n* = 9/18). The earliest reported period of release was 1988 to 2002 (in a study by Kinner et al. 2011) and the study with the most recent year analysed data from 2018 [19]. The studies with the longest release period covered a total of 16 years, which included releases between 2000 and 2015 [20, 21] and the study with the shortest time period was a follow-up of all prisoners released on a specified date in July 2007 [22]. For studies which provided information about incarceration dates (rather than specified release dates), two [23, 24] used a single incarceration date (specified in June 1991) as the index date for follow-up, whereas all other studies used either a single year or range of years. Although some of the key questions in this scoping review were around methodology, the extent to which included studies reported key characteristics varied. For example, in all studies, sex or gender was reported in some format throughout various sections of the paper, whereas age and race or ethnicity were less well documented. Approximately 31% of included studies did not report the age of their study population (*n* = 14) and 31% did not report race or ethnicity in any format (*n* = 14) (Table 2 and appendix 7).
- 2. How is research conducted on this topic?
  The most commonly reported study designs were retrospective cohort studies (*n* = 16), prospective cohort study studies (*n* = 5) and nested case–control studies (*n* = 3) (Table 2). Several included studies did not state the study design (*n* = 7). The type of data used by included studies to investigate prison release and mortality are shown in Table 3. Prison data was often obtained from national prison or criminal registries, department of correction/correctional services or records, or records from single prison systems, for example, individuals released from one county jail. Mortality data used by included studies to determine drug-related death was often obtained from the national death index or national death registries or regional (for example, USA State) death records.
  Study parameters such as number of people released, number of releases (as an individual may have been committed and released more than once during the study period) or person-years of follow-up are reported in Table 2, and included studies differed in size. The study with the largest number of people reported that 229,274 were released over a 16-year time period (between 2000 and 2015) – data from this retrospective cohort study of people released from prison was analysed and presented in two separate papers [25, 26].
  The review found that the terminology that was used in the included studies to report death outcomes varied; the most commonly used terms were overdose deaths, opioid overdose deaths, opioid-related overdose deaths, drug-related deaths and similar, less frequently used variants including death from drug-related infections, drug toxicity and contributing substance use–related cause of death. Approximately 64% of the published studies (*n* = 29) used the codes from the International Classification of Diseases (ICD) to describe cause of mortality (Table 3). Data linkage (or similar meaning terms) was an inclusion criterion in this scoping review. The methods used for data linkage included probabilistic linkage/matching/score (*n* = 17), deterministic linkage (*n* = 2), deterministic and probabilistic linkage (n = 2), personal identifiers or unique identification linkage in methods (*n* = 11), and combinations of name, date of birth, sex or gender and race or ethnicity (*n* = 5). In several studies (*n* = 8) linkage methods were not stated (Table 3).
  Only four studies reported the use of a quality assessment checklist or technique. All four of these studies used the Strengthening the Reporting of Observational Studies in Epidemiology (STROBE) checklist [20, 27–29]. The STROBE guideline provides a checklist of items about the planning and conduct of epidemiological observational studies and best practice requires researchers and authors to include a completed checklist in their reports and papers. Only one study, Chang et al. 2015, provided a copy of the STROBE statement [27].
- 3. What methodologies are used?
  The included studies examined various time periods after release from prison and it was common for studies to examine more than one time period (*n* = 19) (Table 3). Commonly investigated time periods included the first two weeks after release (*n* = 11), the first month (including studies examining intervals up to one month e.g. 1–2 weeks and 3–4 weeks) (*n* = 14) and the first year after release (including studies examining intervals up to one year e.g. up to 4 weeks, after 4 weeks up to 6 months, after 6 months up to 1 year, all follow-up to 1 year) (*n* = 16). During follow-up any re-committals would reduce the at-risk period for mortality as the individual would be in custody rather than in the community, and 60% of included studies took into consideration person-time at risk in the community time and during any subsequent re-incarcerations (*n* = 27). The methods used for dealing with repeated incarcerations included person-time being excluded at re-incarceration i.e. person-time was calculated from the day of release from prison until re-incarceration. Another approach used excluded time during a subsequent incarceration, whereas the time between the next release and death, another incarceration, or the end of the study was included. Other methods used the most recent prison release date/index release was that closest to death or calculated person-time following every release during follow-up until death, re-incarceration or the end of study follow-up. In one study, time periods of 4 weeks and 1 year from the date of first release were used regardless of reimprisonment within these time frames, therefore this method did not exclude time whilst in custody [30]. Another study, coded each re-incarceration after the index release date as an ‘additional post-release booking’ to determine any effect on survival [25].
- 4. What are the findings in relation to mortality?
  A summary of the drug-related mortality outcomes reported in the included studies is provided in appendix 8. Studies reporting SMR by characteristics and time after release are shown in Tables 4 and 5, respectively. CMRs reported by time after release are shown in Table 6. The pooled SMRs across the included studies, grouped by time periods examined after release, are shown in Table 7. The pooled drug-related SMR was 6.99 (95% CI 4.13–11.83; I^2^ = 99.14%) for any time after release (5 studies), 27.07 (95% CI 13.32–55.02; I^2^ = 93.99%) for the first two weeks (4 studies), 10.17 (95% CI 3.74–27.66; I^2^ = 83.83%) for the first 3–4 weeks (3 studies) and 15.58 (95% CI 7.05–34.40; I^2^ = 97.99%) for the first 1 year after release (3 studies) (Table 7). In all studies the SMR was significantly above 1, but in some, this was much higher than others. These results suggest differences in each study. There was a high level of heterogeneity and this must be considered when interpreting the pooled estimates as it may reflect substantial inter-study differences in study design, setting or population. CMRs were not pooled for specific time periods due to a low number of studies reporting these findings. Forest plots are provided in appendix 9. A summary of variables investigated in included studies is provided in appendix 10.
- 5. Where are the knowledge gaps on this topic?
  Our review suggests that knowledge gaps in this topic revolve around methodological differences in study design and limitations in the capacity to synthesise the evidence. Only a limited number of the 45 eligible studies were suitable for inclusion in the pooled analyses for SMRs – there is a need for increased consistency in the use of observational study methodology about mortality among former prisoners. More rigorous reporting of characteristics of former prisoners would allow subgroup analyses to profile those people most at-risk after prison release. For example, reporting characteristics of former prisoners, in terms of age, married or single, health etc. would give a fuller presentation of the results. Our review captured studies from USA, Australia, Canada, Denmark, Norway, Sweden, Taiwan and the UK and pointed to a distinct lack of studies undertaken in low and middle income (LMIC) countries. Clearly, therefore, there is a need for studies to be conducted of this population in LMIC countries in order to understand the extent of global drug-related mortality among people following release from prison.

**Table 2:** Key characteristics of each included study

Journal name	Author [citation]	Year	Reported description of study design	Stated locations	Stated relevant dates	Stated age inclusions (or any stated age exclusions)	Relevant info on study size, number of deaths
Journal of Correctional Health Care	Alex et al. [31]	2017	Quality improvement review process	USA	Deaths occurring from 1 June 2011 to 31 December 2012	Not stated	86,771 discharges; 59 deaths from all-causes [opioid overdose (37.3%); other drug use (8.5%)]
Harm Reduction Journal	Andersson et al. [32]	2020	Retrospective register study	Sweden	Deaths occurring from 1 January 2012 to 31 December 2013 and 1 July 2014 to 30 June 2016	< 65 years	180 deaths by intoxication
American Journal of Geriatric Psychiatry	Barry et al. [33]	2018	Retrospective cohort study	USA	Incarcerated from 2012 to 2014	≥ 50 years	Study: re-entry *n* = 7671 and never incarcerated *n* = 7671. Death by drug overdose: re-entry *n* = 28 and never incarcerated *n* = 10
Drug and Alcohol Dependence	Binswanger et al. [34]	2011	Retrospective cohort study	USA	Released July 1999 to December 2003	Excluded if < 18 years	Cohort: *n* = 30,237 (38,809 releases). Overdose deaths: *n* = 103
Annals of Internal Medicine	Binswanger et al. [35]	2013	Cohort study	USA	Releases between 1 July 1999 and 31 December 2009	Excluded if < 18 years or > 84 years	Cohort: *n* = 76,208 (*n* = 192 511 releases). Overdose mortality (*n* = 558)
Addiction	Binswanger et al. [36]	2016	Nested case control study	USA	Released from July 1999 to December 2009	None stated	All Cause deaths: cases *n* = 699 and controls n = 699. Overdose deaths: cases *n* = 380 and controls *n* = 380
Public Health Reports	Binswanger et al. [18]	2016	Retrospective cohort studies	Australia and USA	Released from 1997 to 2007 (Australia) and 1999 to 2009 (USA)	Australian cohort ≥ 17 years and USA cohort ≥ 18 years	Australian cohort *n* = 69,732 releases, USA cohort *n* = 192,511 releases. All-cause deaths: Australian cohort *n* = 1,563, USA cohort *n* = 2,462. Contributing substance use–related cause of death for any infectious disease as underlying cause: Australian cohort *n* = 14, USA cohort *n* = 49
Addiction	Binswanger et al. [37]	2020	Retrospective cohort study	USA	New sentences from 1 January 2003 to 31 December 2006. Follow-up censored at the time of death or 31 December 2012	≥ 15 years. Juveniles < 18 years and sentenced as children were excluded	Cohort: *n* = 140,266 [sentenced to jail (*n* = 10,788) probation (*n* = 50,202) jail followed by probation (*n* = 54,093) prison (*n* = 24,516) other sentences such as fines or community service (*n* = 656)]. All-cause deaths *n* = 7611 (*n* = 1131 overdoses)
Addiction	Bird et al. [38]	2015	Before and after	UK	Released from 1 January 1996 to 8 October 2007	Not stated. Grouped by 15–34 years and ≥ 35 years	Cohort: *n* = 131,472 (150,517 releases). Drug-related deaths in first 2 weeks *n* = 262 and 12-weeks *n* = 459
Addiction	Bird et al. [39]	2016	Pre–post evaluation of a national policy	UK	Deaths from 2006 to 2010 and 2011 to 2013	Not stated. Grouped by < 35 years and ≥ 35 years	1970 opioid related death (ORDs) in 2006–10; 193 released from prison in the 4 weeks prior to death. 1212 ORDs in 2011–13; 76 released from prison in the 4 weeks prior to death
Drug and Alcohol Dependence	Brinkley-Rubinstein et al. [40]	2018	Not stated (retrospective cohort)	USA	Deaths from 2014 to 2015	≥ 18 years	530 fatal overdoses; 79 had past year incarceration
Jama Network Open	Brinkley-Rubinstein et al. [20]	2019	Retrospective cohort study	USA	Released from 1 January 2000 to 31 December 2015	Not stated	Cohort: 229,274 (398,158 releases). 14,086 deaths after release (1321 opioid overdose deaths)
Addiction	Bukten et al. [41]	2017	Prospective cohort study	Norway	Released from (1 January 2000 to 31 December 2014 and deaths from 2000 to 2014	Not stated	Cohort: 92,663 (153,604 releases). 1–6 months: all-causes deaths *n* = 882, overdose deaths *n* = 493
Journal of Addiction Medicine	Calcaterra et al. [42]	2012	Retrospective cohort study	USA	Released from 1 July 1999 to 31 December 2003	Not stated	Cohort: *n* = 30,237. Deaths: all-causes *n* = 443, Cocaine only-Related Deaths *n* = 49
Lancet Psychiatry	Chang et al. [27]	2015	Nationwide longitudinal cohort study	Sweden	Imprisoned since 1 January 2000 and released before 31 December 2009	Not stated. Table shows ≥ 16 years	Cohort: 47,326. Deaths: all-causes *n* = 2874
Addiction	Degenhardt et al. [43]	2014	Retrospective data linkage study	Australia	Entered OST from 1985 to 2010 and released from 2000 to 2012	Not stated. Median age range of first incarceration 14–64 years	Cohort: 16,453 (60,161 releases). Deaths: all deaths (2000–March 2012) *n* = 1050, accidental drug-induced deaths (2000–10) *n* = 381
Addiction	Forsyth et al. [44]	2014	Retrospective cohort study	Australia	Released from 1 January 1994 to 31 December 2007	Not stated. Table shows 17 to ≥ 60 years	Cohort *n* = 42,015 (82,315 releases). Deaths: all-cause *n* = 2158, drug-related *n* = 450
Addiction	Forsyth et al. [45]	2018	Prospective cohort study	Australia	Recruited within 6 weeks of expected release from August 2008 to July 2010. Censored on 31 May 2013 or death	Not stated. Characteristics reported for < 25 years and > 25 years	Cohort *n* = 1320. Deaths: all-cause *n* = 42 including drug-related *n* = 14
Addiction	Gan et al. [46]	2021	Cohort study	Canada	Released from 1 January 2010 to 31 December 2014 and follow‐up from 1 January 2015 to 31 December 2017	≥ 18 years	Cohort: *n* = 765,690 at baseline, *n* = 5743 incarceration history. Deaths from drug overdose *n* = 634
Scandinavian Journal of Public Health	Gjersing et al. [47]	2013	Retrospective registry study	Norway	Deaths from 1 January 2006 to 31 December 2008 (released up to 6 months before death)	15–65 years	Cohort: *n* = 231. Released from prison within 6 months before death *n* = 18
JAMA Psychiatry	Green et al. [48]	2018	Retrospective cohort analysis	USA	Deaths from 1 January to 30 June 2016 and from 1 January to 30 June 2017 (defined recently incarcerated as 12 months since release)	Not stated. Table shows ≥ 18 years	1 January 2016 to 30 June 2016: *n* = 4005 releases. 2016 period: 26 of 179 overdose deaths were recently incarcerated. 1 January 2017 to 30 June 2017: *n* = 3426 releases. 2017 period: 9 of 157 overdose deaths were recently incarcerated
Plos One	Groot et al. [49]	2016	Descriptive retrospective longitudinal study	Canada	Deaths from 2006 and 2013	≥ 18 years	Cohort: *n* = 6,978 deaths by drug toxicity (*n* = 702 deaths within one year of release)
Drug and Alcohol Dependence	Haas et al. [19]	2021	Retrospective observational case–control study	USA	Released by 30 November 30 2018. Deaths from 1 January 2014 to 31 December 2018. Pilot program jail-based methadone treatment from October 2013 and April 2014	Not stated. Table ≥ 20 years	Cohort: *n* = 1564. Fatal overdoses *n* = 29
Public Health Reports	Hacker et al. [50]	2018	Not stated	USA	Deaths from 2008 to 2014	Not stated. Table shows 0–84 years	Opioid-related overdose deaths *n* = 1399, matched population *n* = 957. *N* = 211 incarcerated in year before death
Drug and Alcohol Dependence	Hakansson et al. [51]	2013	Prospective follow-up study	Sweden	ASI assessments from 2001 to 2006. Deaths until 31 December 2008	20–64 years	Cohort: *n* = 4081 released. Total deaths *n* = 166, accidental overdose *n* = 44, substance-use disorder *n* = 3
Addiction	Huang et al. [22]	2011	Prospective cohort study	Taiwan	Released on 16 July 2007. Follow-up until 31 December 2008	No stated. Table shows ≤ 29 years to ≥ 60 years	Cohort: *n* = 4357. Total deaths *n* = 142, *n* = 48 drug overdose and *n* = 16 drug-related infections
Medical Journal of Australia	Kinner et al. [30]	2011	Not stated	Australia	WA cohort: Released from 1 January 1994 to 31 December 1999. Deaths until to 31 December 2003. NSW cohort: Released from 1 January 1988 to 31 December 2002. Deaths until 31 December 2002	Not stated. Table shows < 25 years, 25–39 years, ≥ 40 years	Total *n* = 50,405, WA cohort: *n* = 16 162 and NSW cohort *n* = 82 650. Total of deaths: WA cohort: 699 and NSW cohort: 4827
Canadian Medical Association Journal Open	Kouyoumdjian et al. [52]	2016	Retrospective cohort study	Canada	In custody in 2000. Deaths until 2012	Not stated. Table shows ≥ 15 years	Cohort: *n* = 48 166. Deaths: all-causes *n* = 4126, overdose *n* = 563
Drug and Alcohol Dependence	Krawczyk et al. [26]	2020	Not stated	USA	Criminal justice records from 2013 to 2016	≥ 18 years	Cohort: *n* = 89,591. Incarceration subgroup *n* = 22,145 (*n* = 73 opioid overdose deaths in subgroup)
Drug and Alcohol Dependence	Larochelle et al. [53]	2019	A retrospective cohort study	USA	Followed from January 2014 to December 2014 or death	≥ 11 years	Cohort 6,717,390 person-years of follow-up. Opioid overdose deaths *n* = 1315
American journal of epidemiology	Lim et al. [54]	2012	Retrospective cohort study	USA	Incarceration from 1 January 2001 to 31 December 2005	16–89 years	Cohort: *n* = 155,272.Deaths *n* = 1,149, drug-related deaths *n* = 219
Lancet HIV	Loeliger et al. [55]	2018	Retrospective cohort	USA	Admitted and released from 1 January 2007 to 31 December 2014. Followed until 31 December 2014 or death	≥ 18 years	Cohort: *n* = 1350. Deaths *n* = 184. For deaths with cause reported (*n* = 170), drug overdose *n* = 26
Addiction	Marsden et al. [28]	2017	Prospective observational cohort study	UK	Recruited from September 2010 to August 2013. Released from September 2010 to October 2014. Follow-up until February 2016	≥ 18 years	Cohort: *n* = 12,260 (15,141 releases). At release, OST exposed (*n* = 8,645) or OST unexposed (*n* = 6,496). First year after release, *n* = 160 deaths, fatal drug-related poisoning *n* = 102
Drug and Alcohol Dependence	Pizzicato et al. [56]	2018	Retrospective cohort study	USA	Released 1 January 2010 to 31 December 2016. Deaths from 1 January 2010 to 31 December 2016	Not stated. Table shows 15–84 years	Cohort: 82,780. Deaths *n* = 2,522, overdose deaths *n* = 837
American Journal of Public Health	Ranapurwala et al. [21]	2018	Retrospective cohort study	USA	Released from 1 January 2000 to 31 December 2015. Death from 1 January 2000 to 31 December 2016	Not stated. Table shows ≥ 18 years	Cohort: *n* = 229,274 (387 913 releases). Out-of-prison deaths *n* = 14,086, opioid overdose deaths *n* = 1329
Annals of Epidemiology	Rosen et al. [57]	2020	Not stated	USA	Released from 1 January 2008 to 30 June 2015. Deaths from 2008 to 2016	≥ 18 years	Cohort: *n* = 111,479. Deaths: all-cause *n* = 3,617, alcohol and Substance-related disorders *n* = 172, opioid poisoning (illicit & prescription) *n* = 460
Jama Psychiatry	Saloner et al. [29]	2020	Predictive modeling study	USA	Records in 2015. Deaths occurring in 2016	18–80 years	Cohort: *n* = 2 294 707. *N* = 1537 released from prison
American Journal of Epidemiology	Spaulding et al. [23]	2011	Not stated	USA	Incarcerated on 30 June 1991. Deaths until 31 December 2006	Not stated	Cohort: *n* = 23,510. Deaths: *n* = 2,650. Out of prison deaths *n* = 2,244. Following Release From Prison, deaths by accidental poisoning *n* = 80
American Journal of Public Health	Spaulding et al. [24]	2015	Cohort Study	USA	Incarcerated on 30 June 1991. Deaths until 2010	Not stated	Cohort: *n* = 23 510. Deaths: total *n* = 3863, accidental poisoning total *n* = 123
Journal of Epidemiology and Community Health	Spittal et al. [58]	2014	Retrospective cohort study	Australia	Released from 1 January 1994 to 31 December 2007. Deaths until 31 December 2007	≥ 17 years	Cohort: *n* = 41,970. Deaths: *n* = 2,158, drug related causes *n* = 396
Epidemiology and Psychiatric Sciences	Spittal et al. [59]	2019	Nested case–control study	Australia	Released from 1 January 1994 to 31 December 2007	Not stated	Cohort: *n* = 286 cases and *n* = 286 controls. Deaths: drug overdose *n* = 93
Australian and New Zealand journal of public health	Van Dooren et al. [60]	2013	Not stated	Australia	Released from 1 January 1994 to 31 December 2007. Deaths in 1996, 2001 and 2006	Not stated. adult prisons: Defined young at index release < 25 years and older at index release ≥ 25 years	Cohort: *n* = 42,015. Deaths: all-causes, young at index release *n* = 92 and older at index release *n* = 271, drug-related, young at index release *n* = 40 and older at index release *n* = 79
Addiction	Victor et al. [25]	2021	Retrospective cohort study	USA	Deaths until 31 December 2007	Not stated	Cohort: *n* = 27,940. Deaths: accidental overdose death *n* = 237
Journal of Affective Disorders	Webb et al. [61]	2013	Nested case–control study	Denmark	Contact with the criminal justice system from 1 January 1980. Suicides from 1994 to 2006	≥ 15 years	Cohort: *n* = 9708 cases and *n* = 188,134 controls. *N* = 9708 suicides; *n* = 6904 men and *n* = 2804 women
Journal of the American Academy of Psychiatry and the Law	Wortzel et al. [62]	2012	Data linkage study	USA	Released from 1999 to 2003	≥ 18 years	Cohort: *n* = 3,806 veterans, compared with *n* = 26,431 nonveterans. Deaths: total all-causes *n* = 443 and total overdose *n* = 103

**Table 3:** Key methodological features of each included (record-linkage study)

Author [citation]	Stated methods for repeated incarcerations	Stated time period examined after prison release	Stated methods of linkage	Stated outcome events or summary measures	Stated sources of data
Alex et al. [31]	Not stated	42 days after release	Probabilistic record linkage	All-cause mortality. No ICD codes	Release records. Bureau of Vital Statistics records. Electronic health records
Andersson et al. [32]	Not stated	Prison contact during year prior to death	Personal identification number linkage	Prison contact during year before death. Death by intoxication. ICD codes	Swedish National Board of Forensic Medicine, regional health care services. Municipal social services. National Prison and Probation Service
Barry et al. [33]	None stated	Most recent prison release until death or study completion	Not stated	Death by drug overdose. ICD-10 codes	Veterans Affairs National Patient Care Database. Centers for Medicare and Medicaid Services data (includes prison admission/release dates). Veterans Affairs Suicide Prevention Applications Network. Veterans Affairs National Suicide Data Repository (SDR) (includes cause-specific death information)
Binswanger 2011 et al. [34]	Person-time at risk in the community; for persons with repeated incarcerations during the study period, the time during a subsequent incarceration was excluded, whereas the time between the next release and death, another incarceration, or the end of the study was included	For early deaths, defined as within 30 days of release from prison	Probabilistic score	All-cause mortality, overdose mortality and early (within 30 days of release) mortality. No ICD codes	Department of Corrections’ records. National Death Index
Binswanger 2013 et al. [35]	The time at risk included time after release and excluded time in prison during any subsequent incarcerations	First month, months 2 to 12, and subsequent months after release	Identities were linked probabilistically	All-cause mortality, 11 causes of death and their subcauses, substance related causes, and the most common substance combinations. ICD-10 codes	Administrative records of the Washington State Department of Corrections. National Death Index
Binswanger 2016 et al. [36]	The index release was that closest to death	Not stated	Matched personal identifiers	All-cause mortality and overdose mortality. No ICD codes	Washington State Department of Corrections. National Death Index
Binswanger 2016 et al. [18]	Excluded data on subsequent person-years in custody for people who were reincarcerated after their first release and deaths in custody	0–14 days, 15–90 days, 91–180 days, > 180 days and entire observation period after each release	Linked personal identifiers probabilistically	Infectious disease–related mortality. ICD-10 codes	Retrospective cohort studies of people released from prison in Queensland and Washington State. National death index
Binswanger 2020 et al. [37]	Not stated	Month after prison, parole, and probation release	Linked identifiers	Overdose mortality. ICD-10 codes	Michigan Department of Corrections administrative databases. National Death Index (NDI)-Plus
Bird 2015 et al. [38]	Calculated person-days at liberty in the first 12 weeks after a qualifying release from the day of release up to the earliest of: date of death, date of re-incarceration for at least 14 days or 12 weeks after the qualifying release date	Risk of DRD in the 12 weeks following release; percentage of these DRDs which occurred during the first 14 days	Not stated	First 2 weeks and 12-week DRD totals. ICD-9 and ICD-10 codes	Linked prisoner-mortality database held at Information Services Division
Bird 2016 et al. [39]	Most recent prison release date	4-week after release	Not stated	Opioid-related deaths (ORDs). No ICD codes	National Records of Scotland official statistics on the number of DRDs. Electronically held Scottish prisoner and morbidity records: Scotland’s Privacy Access Committee, Scottish Prison Service and Disclosure Scotland clearances
Brinkley-Rubinstein 2018 et al. [40]	Not stated	Incarcerated in the year before death	Linked deterministically	Fatal overdose. Fentanyl-related overdose deaths. No ICD codes	RI Office of the Medical Examiner on overdose deaths. Records from RI Department of Corrections
Brinkley-Rubinstein 2019 et al. [20]	Person-time was censored at reincarceration. Person-time was calculated from the day of release from prison until death, reincarceration, or the end of 2016	2 weeks, 1 year and complete follow-up after release	Linkage using last and first names, date of birth, and sex	Opioid overdose death. ICD-10 codes	North Carolina Department of Public Safety (NCDPS). North Carolina death records
Bukten et al. [41]	The time at risk includes only time outside prison; both for individuals with one or repeated incarcerations in the study period, all the time incarcerated was excluded	First week, second week, 3–4 weeks and 2–6 months after release and by three different time intervals of release (2000–04, 2005–09, 2010–14)	Personal identification numbers	All-cause and cause-specific mortality. ICD-10 codes	Norwegian prison registry. Norwegian Cause of Death Registry
Calcaterra et al. [42]	For persons with repeated incarcerations during the study period, the time during a subsequent incarceration was excluded, whereas the time between the next release and death, another incarceration, or the end of the study was included	2-week intervals, weeks 1–2, 3–4, 5–6, 7–8 and all weeks after release	Probabilistic score	Causes-of-death 1) non-cocaine psychostimulants 2) cocaine only and 3) all psychostimulants. ICD-10 codes	Washington State Department of Corrections. National Death Index
Chang et al. [27]	Not stated	Not stated	Unique personal identification numbers	All-cause and external-cause mortality. ICD-10 codes	National Crime Register. National Patient Register, inpatient psychiatric hospital admissions, and outpatient care. Cause of Death Register. Longitudinal Integration Database for Health Insurance and Labour Market Studies. Multi-Generation Register
Degenhardt et al. [43]	Included all eligible prison releases. Person-years at risk accrued during time out of prison (time incarcerated was excluded)	First day, first week, first 2 weeks, month and year following release	Probabilistic linkage	Specific causes of death included accidental drug-induced deaths. ICD-10 codes	Pharmaceutical Drugs of Addiction System (PHDAS). The Reoffending Database (ROD) Department of Corrective Services. National Death Index
Forsyth 2014 et al. [44]	Person-time was calculated from every release during follow-up until death, re-incarceration or the end of study follow-up. Deaths in custody were excluded	Up to 4 weeks, after 4 weeks up to 6 months, after 6 months up to 1 year, all follow-up to 1 year and more than 1 year after a release	Linked probabilistically	Alcohol-related, drug-related, substance-related i.e. drug or alcohol cause of death. ICD-9 and 10 codes	Incarceration data from Corrective Services. National Death Index
Forsyth 2018 et al. [45]	Person-time starting from the date of the first release after baseline interview and censored on 31 May 2013 or death, with any time in prison removed from follow-up time at risk	Not stated	Probabilistic linkage	Drug-related deaths and alcohol and other drug-related deaths. ICD-10 codes	Baseline survey. Prison medical records. Community health records. Correctional records. National Death Index
Gan et al. [46]	Cumulative duration of incarcerations during the follow‐up period was excluded from person‐time of follow‐up	3‐year follow‐up period	Deterministic and probabilistic linkage	Overdose‐related death. ICD-9 or ICD-10 codes	Provincial incarceration records. Linked administrative health data, BC Coroners Service and Vital Statistics Agency. Provincial health insurance data
Gjersing et al. [47]	Not stated	Release up to 6 months before death	For matching purposes, the data included full name, personal identification number, date of birth, date of death, postal code for region of death, residential postal code and whether the person had a post-mortem examination	Drug-induced deaths. No ICD codes	National Cause of Death Registry. Data on toxicology from the Institute of Forensic Medicine at the University of Oslo. Norwegian Correctional Services. Social and health services. Public social services
Green et al. [48]	Not stated	Recently incarcerated defined as death within 12 months of release	Not stated	Overdose death attributed to fentanyl. No ICD codes	Office of State Medical Examiners for deaths. Department of Corrections (RIDOC)
Groot et al. [49]	Not stated	Released within the year before death	Matching names and dates of birth	Drug toxicity deaths. No ICD codes	Deaths from Office of the Chief Coroner. Incarceration records from Correctional Services, both part of the Ontario Ministry of Community Safety and Correctional Services
Haas et al. [19]	Excluded those reincarcerated within five days of release. Any outcomes occurring after reincarceration were not included in analysis	After release until fatal overdose, reincarcerated or study end date (31 December 2018)	Matching primarily on name and date of birth, supplemented with data on race/ethnicity when available	Fatal and non-fatal opioid overdose. No ICD codes	Department of Correction (DoC) records. Deaths from the Connecticut Office of the Chief Medical Examiner (OCME)
Hacker et al. [50]	Defined incarceration as ≥ 1 episode of incarceration ever and in the year before death	Incarceration in the year before death	Matching algorithm, including first and last name, date of birth, social security number, and demographic information	Opioid-related overdose death	Allegheny County Medical Examiner autopsy data. Allegheny County Department of Human Services (ACDHS) Data Warehouse
Hakansson et al. [51]	No access to re-incarcerations and releases	Not stated	Not stated	Causes of death. ICD-10 codes	Database of criminal justice clients with substance use problems. National Causes of Death Register
Huang et al. [22]	In repeat incarcerations during the study period, used the date of release from the last incarceration for the starting point to measure the period from prison release to death	First week after release compared to following 4 weeks after release	Unique ID linkage	All-cause mortality and overdose mortality. ICD-9 codes	National Death Registry. Methadone Maintenance Treatment (MMT) database
Kinner et al. [30]	The date of first release from custody was determined; follow-up periods of 4 weeks and 1 year were used regardless of reimprisonment within these time frames	Four weeks and 1 year	Not stated	Cause of death by drug-related, natural and all other causes. ICD-9 and 10 codes	Australian Bureau of Statistics. Data from two recent Australian record-linkage studies conducted in Western Australia and New South Wales were used. WA cohort: all prisoners released from custody. WA Registrar General’s record of deaths. NSW cohort: all prisoners released from custody. National Death Index
Kouyoumdjian et al. [52]	Not stated	Not stated	Deterministic linkage and probabilistic linkage	Cause of death, deaths due to specific preventable diseases of interest, and certain risk factors. ICD-9 codes	Ontario Ministry of Community Safety and Correctional Services. Registered Persons Database. Mortality data Registrar General Death database
Krawczyk et al. [26]	Not stated	Not stated	Probabilistic matching	Opioid overdose death. No ICD codes for death	Maryland statewide criminal justice records. All-payer hospitalization database. Overdose death records
Larochelle et al. [53]	Not stated	Past 12 months, 0–3 months, 4–12 months and not 0–3 months, 13–24 months and not 0–12 months, 25–36 months and not 0–24 months	Multistage deterministic linkage	Opioid overdose death. No ICD codes	APCD. Registry of Vital Records and Statistics (RVRS). Prescription Monitoring Program (PMP). Acute Care Hospital Case Mix (Case Mix). Massachusetts Ambulance Trip Record Information System (MATRIS). Bureau of Substance Addiction Services’ (BSAS) licensed treatment encounters. Department of Corrections (DOC) and Houses of Corrections (HOC)
Lim et al. [54]	Person-years defined as the number of days during which they were not incarcerated from 2001–2005, including days between each discharge and the subsequent incarceration	1–2 weeks, 3–4 weeks, 5–6 weeks, 7–8 weeks, ≥ 9 weeks after release	Probabilistic matching	Underlying cause of death, drug-related death. ICD-10 codes	Jail records. Death and single-adult homeless registries
Loeliger et al. [55]	Incorporated data across multiple incarcerations during follow-up	Not stated	Not stated	All-cause mortality and drug overdose. ICD-10 codes	Linked pharmacy, custodial, death, case management, and HIV surveillance data from Connecticut Departments of Corrections and Public Health
Marsden et al. [28]	Participants could be recruited on each occasion of incarceration during the recruitment period	First year of release: 1–28 days, 29–121 days and 122–365 days	Not stated	All-cause mortality and drug-related poisoning deaths. ICD-10 codes	Prison National Offender Management Information Service (P-NOMIS). Prison IDTS healthcare provider. Justice Statistics Analytical Services (JSAS database). Office for National Statistics, national deaths register, accessed from the Health and Social Care Information Centre (HSCIC). English National Drug Treatment Monitoring System (NDTMS)
Pizzicato et al. [56]	For multiple incarcerations, the time between subsequent incarcerations was excluded from person time at risk	0–2 weeks, 3–4 weeks and ≥ 5 weeks after release	Matched on name, date of birth, and gender	All-cause, overdose, and non-overdose mortality. No ICD codes	Incarceration records. Philadelphia Department of Prisons (PDP). Medical Examiner’s Office and death certificate records. Death records from the Pennsylvania Department of Health’s Bureau of Vital Records
Ranapurwala et al. [21]	For multiple incarcerations, excluded time in prison	2 weeks, 1 year and complete follow-up after release	Linked using last and first names, date of birth, and sex	Cause of death, opioid overdose death. ICD-10 codes	Prison release data from the NC Department of Public Safety. NC death records from the NC Division of Public Health
Rosen et al. [57]	Release from index incarceration to death, another incarceration or December 31, 201 (which ever occurred first)	Not stated. Until death, censored by reincarceration or study end	Deterministic matching algorithms with probabilistic matching routines	Cause of death. ICD-10 code	Records from the prison system. Death records from the NC State Center for Health Statistics
Saloner et al. [29]	Not stated	Not stated. Released in 2015 and outcomes occurring in 2016	Probabilistic matching	Fatal opioid overdose. ICD-9 and ICD-10 codes nonfatal opioid overdose	All-payer hospital discharges. Prescription drug monitoring program (PDMP). Public-sector specialty behavioral treatment criminal justice records
Spaulding 2011 et al. [23]	Person-time included all time (between incarcerations and following the final incarceration) outside of prison during the study period	0– < 1 month, 1– < 6 months, and 6–12 months after released	Matched on name, Social Security number, age, home address, and known aliases	Cause-specific mortality. No ICD codes	Georgia Department of Corrections (GDC). Georgia Death Registry
Spaulding 2015 et al. [24]	Person-years of follow-up for the total cohort, as well as for each period of observation inside and outside prison. Inside prison considered either during the index incarceration or subsequent reincarceration	In prison (either during the index incarceration or subsequent reincarceration) and during first 2 weeks, second 2 weeks and more than 1 month after released	Probabilistic algorithms	Mortality from liver disease HIV and overdose. ICD-9 or ICD-10 codes	Georgia Department of Corrections Planning and Strategic Management Section. Georgia Death Registry, National Death Index
Spittal 2014 et al. [58]	For repeated incarcerations, subsequent time in prison was excluded	First six months and complete follow-up after release	Probabilistic method and manual review	Cause-specific mortality and drug-related deaths. ICD-9 and ICD-10 codes	Queensland Corrective Services (QCS). National Death Index
Spittal 2019 et al. [59]	Not stated	Not stated	Probabilistic matching and clerical review	Death from external causes, defined as drug overdose, suicide, transport accidents or violence. ICD-9 and ICD-10 codes	Queensland Corrective Services (QCS), Queensland Health. National Death Index
Van Dooren et al. [60]	For subsequent incarcerations, time in prison was deducted from time at risk and deaths in prison were excluded	Censored at death or 365 days after release	Probabilistic matching	Drug‐related deaths, other substance abuse and opioid‐related deaths. ICD-9 and ICD-10 codes	Correctional facilities data. Australian Bureau of Statistics
Victor et al. [25]	Coded each reincarceration between the initial 2017 release date and the 2 years following the initial release date as an ‘additional post-release booking’ to determine the potential effect of re-booking(s) on the hazard rate	First 2 weeks, up to 1 year and 2 years after released	Probabilistic linkage	Accidental fatal overdose. No ICD codes	Administrative records from the Marion County Sheriff’s Office (MCSO). Marion County Coroner’s Office (MCCO)
Webb et al. [61]	Not stated	Contact with the criminal justice system from 1 January 1980. Controls were selected during 1994–2006	Unique Central Person Registration number	Cause-specific mortality including self-poisoning by narcotics & hallucinogens. ICD-10 codes	National Causes of Death Register. National Criminal Register. Psychiatric Central Research Register. Central Population Register and the Integrated Database for Labour Market Research (IDA). IDA database
Wortzel et al. [62]	Person-time at risk in the community excluded time in prison during subsequent incarcerations	Not stated	Matched by first name, last name, sex, birth date (month, day, and year, within one year), and eight of the nine digits in the social security number	All-cause deaths. Deaths from injury by self or others, medical deaths, suicide, alcohol or drug overdose, homicide, cardiovascular disease and cancer. ICD-10 codes	Washington State DOC. Veterans Benefit Administration (VBA) database

**Table 4:** Standardized mortality ratios (SMRs) by characteristics

Study	Reported description	Deaths	Drug-related SMR (95%CI)
**Binswanger et al. 2013 **[35]	Overdose death after release	Observed deaths, *n* = 533 Expected deaths, n = 52	10.33 (9.61–11.10)
**Forsyth et al. 2018 **[45]	Drug-related mortality	Observed deaths, *n* = 14 Expected deaths, *n* = 0.43	32 (19–55)
**Larochelle et al. 2019 **[53]	Fatal opioid overdose		
11–49 years: Release from incarceration in past 12 months		Opioid deaths, *n* = 113	30.3 (24.7–35.9)
≥ 50 years: Release from incarceration in past 12 months		Opioid deaths, *n* = 13	27.8 (12.7–42.9)
Female: Release from incarceration in past 12 months		Opioid deaths, *n* = 39	92.4 (63.4–121)
Male: Release from incarceration in past 12 months		Opioid deaths, *n* = 87	23.0 (18.2–27.9)
**Lim et al. 2012 **[54]	Drug-related death		
Age 16–24 years		Deaths, *n* = 9	2.2 (1.0–4.2)
Age 25–34 years		Deaths, *n* = 31	2.3 (1.5–3.2)
Age 35–44 years		Deaths, *n* = 90	2.1 (1.7–2.6)
Age 45–54 years		Deaths, *n* = 76	2.1 (1.7–2.7)
Age 55–64 years		Deaths, *n* = 11	2.4 (1.2–4.3)
Age 65–89 years		Deaths, *n* = 2	9.5 (1.2–34.4)
Sex: Female		Deaths, *n* = 39	5.9 (4.2–8.1)
Sex: Male		Deaths, *n* = 180	1.9 (1.6–2.2)
Race/ethnicity: Non-Hispanic white		Deaths, *n* = 63	5.2 (4.0–6.6)
Race/ethnicity: Non-Hispanic black		Deaths, n = 81	1.4 (1.1–1.8)
Race/ethnicity: Hispanic		Deaths, *n* = 72	2.4 (1.9–3.0)
Race/ethnicity: Asian		Deaths, *n* = 0	
Race/ethnicity: Other		Deaths, *n* = 3	1.6 (0.3–4.6)
Neighbourhood income: Low		Deaths, *n* = 117	1.7 (1.4–2.0)
Neighbourhood income: Middle		Deaths, *n* = 75	3.4 (2.7–4.3)
Neighbourhood income: High		Deaths, *n* = 27	3.3 (2.2–4.9)
**Pizzicato et al. 2018 **[56]	Overdose deaths		
Overall		Observed deaths, *n* = 837 Expected deaths, *n* = 158	5.29 (4.93–5.65)
Age 15–24		Observed deaths, *n* = 64 Expected deaths, n = 2	37.31 (28.17–46.45)
Age 25–34		Observed deaths, *n* = 257 Expected deaths, n = 39	6.54 (5.74–7.34)
Age 35–44		Observed deaths, *n* = 221 Expected deaths, *n* = 46	4.76 (4.14–5.40)
Age 45–54		Observed deaths, *n* = 193 Expected deaths, *n* = 48	3.99 (3.43–4.56)
Age 55–84		Observed deaths, *n* = 102 Expected deaths, *n* = 23	4.50 (3.63–5.38)
Sex: Female		Observed deaths, *n* = 194 Expected deaths, *n* = 15	12.65 (10.87–14.43)
Sex: Male		Observed deaths, *n* = 643 Expected deaths, *n* = 143	4.50 (4.15–4.84)
Race: White, non-Hispanic		Observed deaths, *n* = 443 Expected deaths, *n* = 39	11.23 (10.19–12.28)
Race: Black, non-Hispanic		Observed deaths, *n* = 256 Expected deaths, *n* = 79	3.25 (2.85–3.65)
Race: Hispanic		Observed deaths, *n* = 129 Expected deaths, *n* = 26	5.52 (4.18–5.93)
Race: Other		Observed deaths, *n* = 9 Expected deaths, *n* = 15	0.62 (0.21–1.02)
**Spaulding et al. 2011 **[23]	Accidental poisoning (drug overdose)	Observed deaths, *n* = 80 Expected deaths, *n* = 23	3.48 (2.76–4.33)

*SMR* Standardized Mortality Ratio, *95%CI* 95% confidence interval

**Table 5:** Standardized mortality ratios (SMRs) by time after release

**Study**	**Reported description**		**Drug-related SMR (95%CI)**
**Groot et al. 2016 **[49]	Drug intoxication death		
In the year after release		All ages: Men—Observed mean annual deaths 72. Expected mean annual deaths 6.9 Women—Observed mean annual deaths 14. Expected mean annual deaths 0.52	11.59 (6.38–16.79)
**Kouyoumdjian et al. 2016 **[52]	Overdose		
First 2 weeks after release		-	56.0 (95% CI 15.3–143.4)
Weeks 2 and 4 after release		-	29.0 (95% CI 3.5–104.8)
**Larochelle et al. 2019 **[53]	Fatal opioid overdose		
Release from incarceration in past 12 months		-	30.0 (24.8–35.3)
Release from incarceration: 0–3 months		-	43.2 (32.6–53.8)
Release from incarceration: 4–12 & NOT 0–3 months		-	21.0 (15.8–26.2)
Release from incarceration: 13–24 & NOT 0–12 months		-	16.6 (12.3–20.9)
Release from incarceration: 25–36 & NOT 0–24 months		-	13.2 (8.9–17.6)
**Lim et al. 2012 **[54]	Drug-related death		
Any time		Deaths, *n* = 219	2.2 (1.9–2.5)
First two weeks after release		Deaths, *n* = 25	8.0 (5.2–11.8)
3–4 weeks after release		Deaths, *n* = 12	4.2 (2.1–7.3)
5–6 weeks after release		Deaths, *n* = 10	3.7 (1.8–6.8)
7–8 weeks after release		Deaths, *n* = 5	2.0 (0.6–4.6)
≥ 9 after release		Deaths, *n* = 167	1.9 (1.6–2.2)
**Pizzicato et al. 2018 **[56]	Overdose deaths		
0–2 weeks after release		Observed deaths, *n* = 107 Expected deaths, *n* = 3	36.91 (29.92–43.90)
3–4 weeks after release		Observed deaths, *n* = 39 Expected deaths, *n* = 3	13.86 (9.51–18.21)
≥ 5 weeks after release		Observed deaths, *n* = 691 Expected deaths, *n* = 153	4.53 (4.19–4.87)
**Ranapurwala et al. 2018 **[21]	Opioid overdose death		
2-weeks after release	All opioids deaths	Observed deaths, *n* = 54 Expected deaths, *n* = 1.3	40.5 (29.7–51.3)
1-year after release		Observed deaths, *n* = 339 Expected deaths, n = 32	10.6 (9.5–11.7)
Complete follow-up		Observed deaths, *n* = 1329 Expected deaths, *n* = 160.9	8.3 (7.8–8.7)
2 weeks after release	Heroin deaths	Observed deaths, *n* = 21 Expected deaths, *n* = 0.28	74.4 (42.6–106.3)
1-year after release		Observed deaths, *n* = 119 Expected deaths, *n* = 6.7	17.7 (14.6–20.9)
Complete follow-up		Observed deaths, *n* = 407 Expected deaths, *n* = 28.5	14.3 (12.9–15.7)
2-weeks after release	Methadone deaths	Observed deaths, *n* = 14 Expected deaths, *n* = 0.42	33.5 (15.9–51.0)
1-year after release		Observed deaths, *n* = 96 Expected deaths, *n* = 10.1	9.5 (7.6–11.5)
Complete follow-up		Observed deaths, *n* = 348 Expected deaths, *n* = 57.7	6.0 (5.4–6.7)
2-weeks after release	Other opioids (commonly prescribed)	Observed deaths, *n* = 19 Expected deaths, *n* = 0.53	35.9 (19.8–52.1)
1-year after release		Observed deaths, *n* = 104 Expected deaths, *n* = 12.7	8.2 (6.6–9.8)
Complete follow-up		Observed deaths, *n* = 457 Expected deaths, *n* = 62.5	7.3 (6.6–8.0)
2-weeks after release	Other synthetic narcotics (e.g. fentanyl)	Observed deaths, *n* = 3 Expected deaths, *n* = 0.24	12.4 (0–26.5)
1-year after release		Observed deaths, *n* = 68 Expected deaths, *n* = 5.8	11.8 (9.0–14.6)
Complete follow-up		Observed deaths, *n* = 314 Expected deaths, *n* = 26.3	11.9 (10.6–13.2)

*SMR* Standardized Mortality Ratio, *95%CI* 95% confidence interval

**Table 6:** Crude mortality rates (CMRs) reported by time after release

Study	Terminology	Drug-related CMR (95%CI)
**Degenhardt et al. 2014 **[43]	Accidental drug-induced deaths	Males 4.2 per 1000 person-years (3.7–4.7) [*n* = 312; PY = 74,631] Females 3.1 per 1000 person-years (2.4–3.9) [*n* = 69; PY = 22,531] Both 3.9 per 1000 person-years (3.5–4.3) [*n* = 381; PY = 97,163]
First day		Males 17.0 per 1000 person-years (2.1–61.3) [n = 2; PY = 118] Females 33.5 per 1000 person-years (0.8–186.7) [*n* = 1; PY = 30] Both 20.3 per 1000 person-years (4.2–59.4) [*n* = 3; PY = 148]
First week		Males 25.8 per 1000 person-years (16.0–39.5) [*n* = 21; PY = 812] Females 24.3 per 1000 person-years (7.9–56.8) [*n* = 5; PY = 206] Both 25.5 per 1000 person-years (16.7–37.4) [*n* = 26; PY = 1,018]
First 2 weeks		Males 21.9 per 1000 person-years (15.3–30.5) [n = 35; PY = 1,595] Females 12.4 per 1000 person-years (4.0–28.9) [*n* = 5; PY = 403] Both 20.0 per 1000 person-years (14.3–27.3) [*n* = 40; *n* = 1,999]
First 4 weeks		Males 16.2 per 1000 person-years (12.0–21.4) [*n* = 50; PY = 3,080] Females 7.7 per 1000 person-years (2.8–16.8) [*n* = 6; PY = 778] Both 14.5 per 1000 person-years (11.0–18.8) [*n* = 56; PY = 3,858]
First year		Males 6.9 per 1000 person-years (5.9–8.0) [*n* = 166; PY = 24,031] Females 4.9 per 1000 person-years (3.3–6.9) [31 6 342] Both 6.5 per 1000 person-years (5.6–7.5) [*n* = 197; PY = 30,373]
**Forsyth et al. 2018 **[45]	Drug-related mortality	3.4 per 1000 person-years (2.0–5.7) [Deaths observed 14; Expected 0.43]
**Spittal et al. 2014 **[58]	Drug-related deaths	14.6 per 10,000 person-years (13.3–16.2)
First 2 weeks after any release		114.0 per 10,000 person-years (70.9–183.4)
Subsequent 24 weeks		27.2 per 10,000 person-years (20.6–35.7)
First six months after any release		33.9 per 10,000 person-years (26.6–43.1)
After first six months		13.2 per 10,000 person-years (11.8–14.7)

*CMR* Crude Mortality Rate, *95%CI* 95% confidence interval

**Table 7:** Pooled standardized mortality ratios (SMRs) across the included studies, grouped by time periods examined after release

Time after release	Number of studies	Pooled measure (95%CI)	Heterogeneity I^2^
**Standardised mortality ratios**			
Any time	5	6.99 (4.13–11.83)	99.14%
First 2 weeks	4	27.07 (13.32–55.02)	93.99%
First 3–4 weeks	3	10.17 (3.74–27.66)	83.83%
First year	3	15.58 (7.05–34.40)	97.99%

## Discussion

This scoping review maps and summarises research evidence from record linkage studies about drug-related deaths among former adult prisoners and the extent to which drug-related causes contribute to post-release prisoner mortality. The research questions in this review focused on the scope of the literature, methodologies used in observational data-linkage studies and the most recent findings in relation to mortality (published between 2011 and 2021). This scoping review found an increased risk of drug-related death after release from prison, particularly in the first two weeks after release, although the drug-related mortality risk remained elevated for the first year among former prisoners. However, despite this review identifying 45 relevant publications, only a limited number of studies were included in the pooled analyses for SMRs due to differences in study design (for example, time periods examined after release) and methodologies used which has significantly limited evidence synthesis. In addition, we found high levels of heterogeneity in our pooled analyses meaning that our interpretation of the pooled estimates is more hesitant.

The findings of our scoping review are supported by previous literature mapping this topic. A recent scoping review by Mital et al. described the relationship between incarceration history (custody in a jail or prison facility) and opioid overdose in North America, including 18 studies published between 2001 and 2019, with the scoping review methodology following guidance by Levac et al. [63, 64]. The review reported four important findings; (1) an increased risk of opioid overdose among formerly incarcerated people; (2) an increased risk of opioid overdose was associated with some demographic, substance use, and incarceration-related characteristics (including substance use disorders and mental health issues); (3) incarceration history was identified as a risk factor for opioid overdose among individuals who inject opioids and (4) opioid overdose was suggested as the leading cause of death in people who have been formerly incarcerated [63].

The results of this review in terms of an increased mortality risk after prison release concurs with the findings previously published in systematic reviews and meta-analyses. It is concerning that post-release mortality risk is high. Collectively, the reviews appear to indicate that post-release mortality has persisted over time. For example, a previous systematic review pooled SMRs from studies which used record linkage methods to examine deaths in ex-prisoners between 1998 and 2011, reporting SMRs for drug-related death of 32.2 (95% CI 22.8–45.4) for < 1 year, 26.2 (95% CI 6.4–107.3) for ≥ 1 year and 27.3 (95% CI 9.8–76.0) for any time after release [6]. A separate systematic review of publications between 1980 and 2011 explored the literature on studies of mortality in released prisoners using linkage of prisoner and mortality databases, and reported all-cause SMR, ranging from 1.0 to 9.4 in males and from 2.6 to 41.3 in females [5]. Furthermore, similar to our findings, where the drug-related death risk was highest in the first two weeks after release; a meta-analysis of mortality during the 12 weeks after prison release reported an increased risk of drug-related mortality during the first 2 weeks after prison release compared to the subsequent 10 weeks (however, the mortality risk was elevated during the first 4 weeks) [7].

Kinner et al., Merrall et al. and Zlodre and Fazel, all reported high levels of heterogeneity, for example between countries [7], in study design [5, 6], and in analysis and findings of publications [6]. In our scoping review, differences in the study design, methodologies and findings of included studies limited the degree to which studies could be synthesised meaningfully. The included studies examined various time periods after release from prison and this limited the number of studies included in the pooled analyses in this review. Differences were also found in study design, i.e. retrospective cohort, prospective cohort and nested case–control study designs, but differences were also found in methodologies, for example in the approaches used for determining the time at-risk during follow-up. During re-incarceration, re-committals would reduce the at-risk period for drug-related mortality as the individual would be in custody rather than in the community. Other differences included various types of data used by included studies to determine mortality (for example, national and regional death records) and prison release (for example, national prison registries and single prison records). The type and geographical distribution of death records used in the study would likely have affected the number of missed deaths, for example if mortality records covered one country and the death occurred outside of this border. The study size differed in the included publications and the size of the prison population(s) and location(s) of prison(s) would affect the generalisability of the study findings. The terminology used to describe or define drug-related deaths differed between studies, with some studies using ICD codes and definitions. The definition used to describe drug-related deaths may have an effect on the findings, for example combining multiple ICD codes for drug-related deaths in the definition would be more inclusive compared to very specific definitions.

The reporting of characteristics of individuals varied between included studies. Gender/sex was reported in all studies, but age and race/ethnicity were only reported in one-third of papers, making it difficult to contextualise the findings. Approximately 9% of included studies stated the use of a quality assessment checklist or technique and in only one study was a copy of the STROBE statement provided as an appendix. Adequate reporting of research facilitates the assessment of published studies and following recommended guidelines in the reporting of research allows rigour and transparency in the process. In summary, this review suggests a need for a more consistent methodology and rigorous reporting of observational studies about mortality among former prisoners.

## Study strengths and limitations

Although meta-analyses are not consistent with the methodology of scoping reviews [10, 11], this scoping review included exploratory meta-analyses. We conducted a scoping review (rather than a systematic review) because we wanted to scope and search broadly and at the same time deepen the level of critical analysis where there was an opportunity to do so. For example, the review included a focus on how record linkage research was conducted, and on differences in methodologies that were used among record-linkage studies in this research area. This broader focus stemmed largely from the results of previous systematic reviews/meta-analysis that reported high levels of heterogeneity [5–7]. Our scoping review summarised the methodologies and findings narratively, and the accompanying meta-analyses added to this narrative by explaining high levels of heterogeneity in our pooled analyses, and showing differences across studies. This review recommends that a more consistent approach to methodology and reporting is followed in the future. This scoping review has several strengths. This is the first scoping review of record linkage studies about drug-related deaths among former adult prisoners. The methods followed the first five stages of the framework for conducting scoping reviews by Arksey and O’Malley [10] and adhered to the guidance developed by the Joanna Briggs Institute (JBI) and the JBI Collaboration. The methods for this scoping review were previously published in a protocol allowing transparency and forward planning [9]. Modifications from the original protocol have been stated in this review, and in a deviation from that stated in the protocol, data were independently extracted by one reviewer, with a proportion of papers checked by using a second reviewer due to time constraints. Using this approach allowed a check of the accuracy and consistency of the recorded information. There are some limitations to this review, the search strategy was limited to publications available in English due to resources for translation and the review did not include a search of the grey literature which may limit the interpretation of the findings.

## Future research and policy

This scoping review focused on former prisoners. However reviews on other prisoner groups, such as prisoners on remand or probation, would be of benefit. Prisoners have higher rates of mental and physical health problems compared to the general population, and substance use disorders are common in people who are committed to prison. Research on mental and physical health conditions, substance use disorders, and physical and mental ill health comorbidity in people released from prison could help profile risk after release. As part of this scoping review process, authors identified one randomised controlled trial in Australia and one randomized controlled pilot trial after prison release in England, but these publications were excluded at the full screening stage [65, 66]. The NALoxone InVEstigation (N-ALIVE) pilot trial tested feasibility measures for randomized provision of naloxone-on-release to eligible prisoners and demonstrated the feasibility of recruiting prisons and consenting of prisoners [65]. A randomised controlled trial of a service brokerage intervention for adult former prisoners involved an intervention group receiving a personalised booklet with their health status and appropriate community health services, and telephone contact for each week in the first month after release to assess any health needs and health service utilisation (control arm received usual care) [66]. A separate review of trials in former prisoners after release would provide evidence to help guide the development of future research in this area.

This review was undertaken in response to concerns from public health, criminal justice, voluntary and community groups and wider society about prisoner health and well-being in Northern Ireland after release from prison. Our findings suggest the need for formalised joined-up working and interagency collaboration regarding the way in which people released from prison are supported, and an ongoing review and consideration of interventions and service responses designed to reduce drug-related deaths among this group, including novel service responses such as overdose centres, transition clinics and drug consumption rooms [67, 68]. It is clear from the available evidence that the transition from prison to community is an at-risk period and there is need for sustained joined-up service responses and support that help people released from prison to negotiate this transition.

## Conclusions

This scoping review found an increased risk of drug-related death after release from prison, particularly in the first two weeks after release, although the drug-related mortality risk remained elevated for the first year among former prisoners. Our results are of concern as we show that post-release mortality risk is still high despite similar findings having been reported in the literature more than a decade ago. This scoping review has detailed examples of differences in study design and methodology in included studies which has significantly limited evidence synthesis. This review suggests a need for a more consistent methodology and rigorous reporting of observational studies about mortality among former prisoners.

## Supplementary Information

**Additional file 1.** 

**Additional file 2.** 

## Data Availability

Our submitted paper is a review and does not contain raw data in the usual meaning of that term. However, we have included, as a supplementary file, a data charting form showing all data fields that were extracted from the full texts of eligible papers.

## Abbreviations

CMOs: Chief Medical Officers
CIs: Confidence intervals
CMRs: Crude mortality rates
ICD: International Classification of Diseases
JBI: Joanna Briggs Institute
PRISMA-ScR: Preferred Reporting Items for Systematic reviews and Meta-Analyses extension for Scoping Reviews
SE: Standard Error
SMRs: Standardised mortality ratios
STROBE: Strengthening the Reporting of Observational Studies in Epidemiology
