# Supplementary material for: Record linkage studies of drug-related deaths among adults who were released from prison to the community: a scoping review
Source: BMC Public Health. 2023 May 5;23:826. doi: 10.1186/s12889-023-15673-0 (PMC10161544; doi:10.1186/s12889-023-15673-0)
Supplement: Supplementary file 2 — Additional file 2. [file 12889_2023_15673_MOESM2_ESM.docx]

**Record linkage studies of drug-related deaths among adults who were released from prison to the community: a scoping review**

**Authors**

Janine A. Cooper ^1,2^*, Ifeoma Onyeka ^1,2^^, Christopher Cardwell ^1^, Euan Paterson ^1,2^, Richard Kirk ^3^, Dermot O’Reilly ^1,2^, Michael Donnelly ^1,2^

^1^ Centre for Public Health, Queen's University Belfast, Royal Hospitals Site, Grosvenor Road, Belfast, UK

^2^ Administrative Data Research Centre Northern Ireland (ADRC NI), Centre for Public Health, Queen's University Belfast, Royal Hospitals Site, Grosvenor Road, Belfast, UK

^3^ South Eastern Health and Social Care Trust, Ulster Hospital, Dundonald, UK

^ Present address: Sheffield Hallam University, Collegiate Crescent Campus, Sheffield, UK

*corresponding author

**Appendix 1 Search strategy has been developed for MEDLINE**

Ovid MEDLINE(R) ALL

1 Prisoners/

2 ex-prisoner*.mp.

3 former prisoner*.mp.

4 inmate*.mp.

5 ex-inmate*.mp.

6 former inmate*.mp.

7 Criminals/

8 ex-criminal*.mp. [mp=title, abstract, original title, name of substance word, subject heading word, floating sub-heading word, keyword heading word, organism supplementary concept word, protocol supplementary concept word, rare disease supplementary concept word, unique identifier, synonyms]

9 former criminal*.mp.

10 convict*.mp.

11 ex-convict*.mp.

12 former convict*.mp.

13 detainee*.mp.

14 ex-detainee*.mp.

15 former detainee*.mp.

16 Prisons/

17 Jails/

18 gaol*.mp.

19 imprison*.mp.

20 incarcerat*.mp. [mp=title, abstract, original title, name of substance word, subject heading word, floating sub-heading word, keyword heading word, organism supplementary concept word, protocol supplementary concept word, rare disease supplementary concept word, unique identifier, synonyms]

21 detention*.mp.

22 correction*.mp.

23 confinement.mp.

24 Correctional Facilities/

25 penal institution*.mp.

26 penitentiar*.mp. [mp=title, abstract, original title, name of substance word, subject heading word, floating sub-heading word, keyword heading word, organism supplementary concept word, protocol supplementary concept word, rare disease supplementary concept word, unique identifier, synonyms]

27 remand.mp.

28 offender*.mp.

29 ex-offender*.mp.

30 former offender*.mp.

31 after release.mp.

32 prison release.mp.

33 released prisoner*.mp.

34 following release.mp.

35 recently released.mp.

36 newly released.mp.

37 postrelease.mp.

38 post-release.mp.

39 liberat*.mp.

40 "Cause of Death"/ or Death/

41 Mortality/

42 Fatal Outcome/

43 fatal*.mp.

44 lethal*.mp.

45 Substance-Related Disorders/

46 Opioid-Related Disorders/

47 drug abuse*.mp.

48 drug dependen*.mp.

49 Drug Misuse/ or Prescription Drug Misuse/ or Substance Abuse, Intravenous/

50 Drug Overdose/

51 drug poisoning.mp.

52 Drug Users/ or Injections, Intravenous/

53 substance abuse*.mp.

54 substance dependen*.mp.

55 substance misuse*.mp.

56 substance use*.mp.

57 Illicit Drugs/

58 substance poisoning.mp.

59 street drug*.mp.

60 toxicity.mp.

61 Opiate Overdose/ or Analgesics, Opioid/

62 Heroin Dependence/ or Heroin/

63 drug withdrawal.mp.

64 accidental poisoning.mp.

65 Benzodiazepines/

66 Cocaine-Related Disorders/ or Cocaine/ or Crack Cocaine/ or Cocaine Smoking/

67 Narcotics/

68 1 or 2 or 3 or 4 or 5 or 6 or 7 or 8 or 9 or 10 or 11 or 12 or 13 or 14 or 15 or 16 or 17 or 18 or 19 or 20 or 21 or 22 or 23 or 24 or 25 or 26 or 27 or 28 or 29 or 30 or 31 or 32 or 33 or 34 or 35 or 36 or 37 or 38 or 39

69 40 or 41 or 42 or 43 or 44

70 45 or 46 or 47 or 48 or 49 or 50 or 51 or 52 or 53 or 54 or 55 or 56 or 57 or 58 or 59 or 60 or 61 or 62 or 63 or 64 or 65 or 66 or 67

71 68 and 69 and 70

72 limit 71 to english language

73 limit 72 to yr="2011 -Current"

**Appendix 2 Search strategy has been developed for EMBASE**

Embase

1 prisoner/

2 ex-prisoner*.mp.

3 former prisoner*.mp.

4 inmate*.mp.

5 ex-inmate*.mp.

6 former inmate*.mp.

7 criminal*.mp.

8 ex-criminal*.mp. [mp=title, abstract, heading word, drug trade name, original title, device manufacturer, drug manufacturer, device trade name, keyword heading word, floating subheading word, candidate term word]

9 former criminal*.mp.

10 convict*.mp.

11 ex-convict*.mp.

12 former convict*.mp.

13 detainee*.mp.

14 ex-detainee*.mp.

15 former detainee*.mp.

16 prison/

17 jail*.mp.

18 gaol*.mp.

19 imprison*.mp.

20 incarcerat*.mp.

21 detention/

22 correction*.mp.

23 confinement.mp.

24 correctional facility/

25 penal institution*.mp.

26 penitentiar*.mp.

27 remand.mp.

28 offender/

29 ex-offender*.mp.

30 former offender*.mp.

31 after release.mp.

32 prison release.mp.

33 released prisoner*.mp.

34 following release.mp.

35 recently released.mp.

36 newly released.mp.

37 postrelease.mp.

38 post-release.mp.

39 liberat*.mp.

40 death/ or "cause of death"/

41 mortality/

42 fatality/

43 fatal outcome*.mp.

44 fatal*.mp.

45 lethal*.mp.

46 substance-related disorder*.mp.

47 opioid-related disorder*.mp.

48 drug abuse/

49 drug dependence/

50 drug misuse/

51 prescription drug misuse/

52 substance abuse/

53 drug overdose/

54 drug poisoning.mp.

55 Drug Users.mp.

56 "drug use"/

57 intravenous drug administration/

58 substance dependen*.mp.

59 substance misuse*.mp.

60 substance use*.mp.

61 illicit drug/

62 substance poisoning.mp.

63 street drug/

64 toxicity/ or "drug toxicity and intoxication"/ or drug toxicity/

65 Opiate Overdose.mp.

66 opiate/

67 heroin dependence/

68 Heroin.mp.

69 drug withdrawal/

70 accidental poisoning.mp.

71 benzodiazepine/

72 cocaine dependence/ or cocaine/ or cocaine smoking/

73 cocaine-related disorder*.mp.

74 narcotic*.mp.

75 1 or 2 or 3 or 4 or 5 or 6 or 7 or 8 or 9 or 10 or 11 or 12 or 13 or 14 or 15 or 16 or 17 or 18 or 19 or 20 or 21 or 22 or 23 or 24 or 25 or 26 or 27 or 28 or 29 or 30 or 31 or 32 or 33 or 34 or 35 or 36 or 37 or 38 or 39

76 40 or 41 or 42 or 43 or 44 or 45

77 46 or 47 or 48 or 49 or 50 or 51 or 52 or 53 or 54 or 55 or 56 or 57 or 58 or 59 or 60 or 61 or 62 or 63 or 64 or 65 or 66 or 67 or 68 or 69 or 70 or 71 or 72 or 73 or 74

78 75 and 76 and 77

79 limit 78 to english language

80 limit 79 to yr="2011 -Current"

**Appendix 3 Search strategy has been developed for PsychINFO**

APA PsycInfo

1 exp Prisoners/

2 ex-prisoner*.mp.

3 former prisoner*.mp.

4 inmate*.mp.

5 ex-inmate*.mp.

6 former inmate*.mp.

7 exp Criminal Offenders/

8 criminal*.mp.

9 ex-criminal*.mp.

10 former criminal*.mp.

11 convict*.mp.

12 ex-convict*.mp.

13 former convict*.mp.

14 detainee*.mp.

15 ex-detainee*.mp.

16 former detainee*.mp.

17 exp Prisons/

18 jail*.mp.

19 gaol*.mp.

20 imprison*.mp.

21 exp Incarceration/

22 incarcerat*.mp. [mp=title, abstract, heading word, table of contents, key concepts, original title, tests & measures, mesh word]

23 exp Legal Detention/

24 detention*.mp.

25 exp Correctional Institutions/

26 correction*.mp.

27 confinement.mp.

28 correctional facilit*.mp. [mp=title, abstract, heading word, table of contents, key concepts, original title, tests & measures, mesh word]

29 penal institution*.mp.

30 penitentiar*.mp.

31 remand.mp.

32 exp Female Criminal Offenders/ or exp Mentally Ill Offenders/ or exp Male Criminal Offenders/

33 offender*.mp.

34 ex-offender*.mp.

35 former offender*.mp.

36 after release.mp.

37 prison release.mp.

38 released prisoner*.mp.

39 following release.mp.

40 recently released.mp.

41 newly released.mp.

42 postrelease.mp.

43 post-release.mp.

44 liberat*.mp. [mp=title, abstract, heading word, table of contents, key concepts, original title, tests & measures, mesh word]

45 exp "Death and Dying"/

46 death.mp.

47 cause of death.mp.

48 mortality.mp.

49 fatal outcome.mp.

50 fatal*.mp.

51 lethal*.mp.

52 exp "Substance Use Disorder"/

53 substance-related disorder*.mp.

54 exp "Opioid Use Disorder"/

55 opioid-related disorder*.mp.

56 exp Drug Abuse/

57 exp Drug Dependency/

58 drug dependen*.mp. [mp=title, abstract, heading word, table of contents, key concepts, original title, tests & measures, mesh word]

59 exp Prescription Drug Misuse/

60 drug misuse.mp.

61 substance abuse.mp.

62 exp Intravenous Injections/ or exp Intravenous Drug Usage/

63 exp Drug Overdoses/

64 drug poisoning.mp.

65 exp Drug Usage/

66 drug user*.mp.

67 substance abuse*.mp.

68 substance dependen*.mp.

69 substance misuse*.mp.

70 substance use*.mp.

71 illicit drug*.mp.

72 substance poisoning.mp.

73 street drug*.mp.

74 exp Toxicity/

75 exp Opiates/

76 opiate overdose.mp.

77 exp Heroin Addiction/ or exp Heroin/

78 heroin dependen*.mp.

79 exp Drug Withdrawal/

80 accidental poisoning.mp.

81 exp Benzodiazepines/

82 exp Crack Cocaine/ or exp Cocaine/

83 cocaine-related disorder*.mp.

84 cocaine smoking.mp.

85 exp Narcotic Drugs/

86 1 or 2 or 3 or 4 or 5 or 6 or 7 or 8 or 9 or 10 or 11 or 12 or 13 or 14 or 15 or 16 or 17 or 18 or 19 or 20 or 21 or 22 or 23 or 24 or 25 or 26 or 27 or 28 or 29 or 30 or 31 or 32 or 33 or 34 or 35 or 36 or 37 or 38 or 39 or 40 or 41 or 42 or 43 or 44

87 45 or 46 or 47 or 48 or 49 or 50 or 51

88 52 or 53 or 54 or 55 or 56 or 57 or 58 or 59 or 60 or 61 or 62 or 63 or 64 or 65 or 66 or 67 or 68 or 69 or 70 or 71 or 72 or 73 or 74 or 75 or 76 or 77 or 78 or 79 or 80 or 81 or 82 or 83 or 84 or 85

89 86 and 87 and 88

90 limit 89 to english language

91 limit 90 to yr="2011 -Current"

**Appendix 4 Search strategy has been developed for Web of Science**

Web of Science

prisoner* or “ex-prisoner*” or “former prisoner*” or inmate* or “ex-inmate*” or “former inmate*” or criminal* or “ex-criminal*” or “former criminal*” or convict* or “ex-convict*” or “former convict*” or detainee* or “ex-detainee*” or “former detainee*” or prison* or jail* or gaol* or imprison* or incarcerat* or detention* or correction* or confinement or “correctional facilit*” or “penal institution*” or penitentiar* or remand or offender* or “ex-offender*” or “former offender*” or “after release” or “prison release” or “released prisoner*” or “following release” or “recently released” or “newly released” or postrelease or “post-release” or liberat* (All Fields) and "cause of death" or death or mortality or “fatal outcome” or fatal* or lethal* (All Fields) and “substance-related disorder*” or “opioid-related disorder*” or “drug abuse*” or “drug dependen*” or “drug misuse” or “prescription drug misuse” or intravenous or “drug overdose” or “drug poisoning” or “drug use*” or injection* or “substance abuse*” or “substance dependen*” or “substance misuse*” or “substance use*” or “illicit drug*” or “substance poisoning” or “street drug*” or toxicity or “opiate overdose” or opioid or “heroin dependen*” or heroin or “drug withdrawal” or “accidental poisoning” or benzodiazepine* or “cocaine-related disorder*” or cocaine or “crack cocaine” or “cocaine smoking” or narcotic* (All Fields) and English (Languages) and 2011 or 2012 or 2013 or 2014 or 2015 or 2016 or 2017 or 2018 or 2019 or 2020 or 2021 (Publication Years)

**Appendix 5 Data charting form**

**Record linkage studies of drug-related deaths among former adult prisoners** **who have been released to the community: a scoping review**

**DATA CHARTING FORM**

| **Study assessor initials** | |  |
| --- | --- | --- |
| **Date of completion of data charting form** | |  |
|  | | |
| **Item** | ***Notes on item*** | **Data extracted** |
| **Study reference number** |  |  |
| **Study assessment number** |  |  |
| **Lead author** |  |  |
| **Title** |  |  |
| **Year of publication** |  |  |
| **Origin, country of origin** |  |  |
| **Journal name** |  |  |
| **Study design** |  |  |
| **Citation** |  |  |
| **Setting** | *Stated setting* |  |
|  | *Stated locations* |  |
|  | *Stated relevant dates* |  |
| **Participants** | *Stated study population* |  |
|  | *Stated type of prison* |  |
|  | *Stated age inclusions (or any stated age exclusions)* |  |
|  | *Stated gender inclusions* |  |
|  | *Stated race/ethnicity inclusions* |  |
|  | *Stated methods of study population selection (type of prisoner data records)* |  |
|  | *Stated methods of follow-up (for example, record linkage or interview)* |  |
|  | *Stated rationale for the choice of cases and controls (case-control study only)* |  |
|  | *Stated matching criteria (case-control study only)* |  |
|  | *Stated number of controls per case (case-control study only)* |  |
| **Data sources** | *Stated sources of data* |  |
|  | *Stated sources of comparator data* |  |
| **Bias** | *Stated bias* |  |
|  | *Stated efforts to address potential sources of bias* |  |
|  | *Stated methods for dealing with repeated incarcerations?* |  |
| **Study size** | *Stated study size* |  |
| **Statistical methods** | *Stated statistical methods* |  |
|  | *Stated methods controlling for confounders and methods for examining subgroups and interactions* |  |
|  | *Stated sensitivity analyses* |  |
|  | *Stated time period examined after prison release* |  |
|  | *Stated how missing data was addressed* |  |
|  | *Stated how loss to follow-up was addressed* |  |
|  | *Stated how matching of case and controls was addressed (case-control study only)* |  |
| **Linkage** | *Stated linkage i.e. if person-level, institution-level or other data linkage across two or more databases* |  |
|  | *Stated types of linked databases* |  |
|  | *Stated methods of linkage* |  |
| **Main outcomes** | *Stated outcome events or summary measures*  *(include information on specific drugs)* |  |
| **Main results** | *Stated unadjusted estimate and, if applicable, confounder adjusted estimates, their precision, which confounders were adjusted for.*  *Reported for all cause and drug-related deaths*  *For example, include crude mortality rates, standardised mortality ratios etc.*  *Include reported for age, gender, race/ethnicity etc* |  |
|  | *Stated age at release (mean/median/SD/range)* |  |
|  | *Stated length of incarceration (mean/median/SD/range)* |  |
| **Limitations** | *Stated limitations of study including sources of potential bias or imprecision (are the results generalisable)* |  |
| **Quality assessment checklist or technique** | *Stated if quality assessment checklist or technique was used* |  |

**Appendix 6 Distribution of included studies**

**Geographical distribution of studies**

**Number of publications included in scoping review per year**

**Appendix 7 Table characteristics of each included study (stated gender and race/ethnicity)**

| **Author [citation]** | **Gender inclusions** | **Race/ethnicity inclusions** |
| --- | --- | --- |
| Alex et al. [18] | Male/Female | Race/ethnicity |
| Andersson et al. [19] | Table reported gender (Male) | Not reported |
| Barry et al. [20] | Male/Female | Race |
| Binswanger 2011 et al. [21] | Male/Female | Race/ethnicity |
| Binswanger 2013 et al. [22] | Male/Female | Race/ethnicity |
| Binswanger 2016 et al. [23] | Men/Women | Race/ethnicity |
| Binswanger 2016 et al. [24] | Male/Female | Racial/ethnic minority group |
| Binswanger 2020 et al. [25] | Male/Female | Race |
| Bird 2015 et al. [26] | Stated 7% Female | Not stated |
| Bird 2016 et al. [27] | Male/Female | Not stated |
| Brinkley-Rubinstein 2018 et al. [28] | Male/Female | Race |
| Brinkley-Rubinstein 2019 et al. [29] | Male/Female | Race |
| Bukten et al. [30] | Men/Women | Not stated |
| Calcaterra et al. [31] | Male/Female | Race and ethnicity |
| Chang et al. [32] | Figure reported Male/Female | Not stated |
| Degenhardt et al. [33] | Table reported Male/Female | Indigenous |
| Forsyth 2014 et al. [34] | Table reported Male/Female | Indigenous status |
| Forsyth 2018 et al. [35] | Table reported Male (78.9%) | Indigenous status |
| Gan et al. [36] | Table reported Male/Female | Not stated |
| Gjersing et al. [37] | Table reported Men/Women | Not stated |
| Green et al. [38] | Table reported Male/Female | Race/ethnicity |
| Groot et al. [39] | Table reported Male/Female | None stated |
| Haas et al. [40] | Male | Race/ethnicity |
| Hacker et al. [41] | Male/Female | Race |
| Hakansson et al. [42] | Males/Females | Not stated |
| Huang et al. [43] | Table reported Male/Female | Not stated |
| Kinner et al. [44] | Men/Women | Indigenous status |
| Kouyoumdjian et al. [45] | Table reported Men/Women | Race |
| Krawczyk et al. [46] | Male/Female | Race |
| Larochelle et al. [47] | Male/Female | Not stated |
| Lim et al. [48] | Male/Female | Race/ethnicity |
| Loeliger et al. [49] | Male/Female | Race/ethnicity |
| Marsden et al. [50] | Male/Female | Not stated |
| Pizzicato et al. [51] | Male/Female | Race/ethnicity |
| Ranapurwala et al. [52] | Table reported Male/Female | Race |
| Rosen et al. [53] | Table reported Male/Female | Race |
| Saloner et al. [54] | Male/Female | Not stated |
| Spaulding 2011 et al. [55] | Sex-race group | Sex-race group |
| Spaulding 2015 et al. [56] | Male/Female | Race/ethnicity |
| Spittal 2014 et al. [57] | Male/Female | Indigenous Australian(N/Y) |
| Spittal 2019 et al. [58] | Men/Women | Indigenous |
| Van Dooren et al. [59] | Male/Female | Indigenous status: Indigenous Australian (%) |
| Victor et al. [60] | Male/Female | Race |
| Webb et al. [61] | Men/Women | Not stated |
| Wortzel et al. [62] | Male/Female | Race or ethnic group |

**Appendix 8 Summary of drug-related mortality outcomes in included studies**

| **Author [citation]** | **Reported for all cause and drug-related deaths. For example, include crude mortality rates, standardised mortality ratios etc.** |
| --- | --- |
| Alex et al. [18] | Post-release all-cause mortality rate was 5.89 per 1,000 person-years. 86,771 discharges; 59 deaths occurred within 42 days after release. In the first 42 days, causes of death included opioid overdose (37.3%) and other drug use (8.5%) with the average number of days after release before death occurred was 18 days for both groups. |
| Andersson et al. [19] | Prison contact during the year before to death (percentage of total deaths n=180); 4.9% Methadone (n = 82) (reference); 6.7% Buprenorphine (n = 30); 12.5% Heroin (n = 40); 10.7% Fentanyl (n = 28); 7.8% Total (n = 180). |
| Barry et al. [20] | Older re-entry veterans had significantly higher rates of death by drug overdose compared with veterans never incarcerated (121.7 versus 43.5; adjusted HR 3.45; 95%CI 1.37–8.73). Adjusted for homelessness, sum of 13 medical conditions, traumatic brain injury, and any psychiatric disorder. |
| Binswanger 2011 et al. [21] | Overdose mortality rate was 181 per 100,000 person-years; 95%CI 149-219. Of the 103 overdose deaths, 32 (31%) were within 30 days. In those released <50 years, the hazard of overdose death was 93% higher for each additional decade of age [hazard for mortality decelerated after age 50 (not statistically significant)]. Increasing years incarcerated was associated with a reduced hazard of overdose mortality (HR 0.80 for each additional year; 95%CI 0.68-0.95). |
| Binswanger 2013 et al. [22] | Overdose mortality rate after release was 167 per 100,000 person-years; 95% CI 153-181 (n = 558).  Overdose mortality rate after release (Men) was 154 per 100,000 person-years; 95% CI 140–169 (n=436).  Overdose mortality rate after release (Women) was 236 per 100,000 person-years; 95% CI 194–277 (n=122).  Multivariable models - increased risk for overdose (adjusted HR 1.38; 95% CI 1.12-1.69) and opioid-related deaths (adjusted HR 1.39; 95% CI 1.09-1.79) for females after release. Overdose after release SMR 10.33; 95% CI 9.61–11.10. In multivariable models, the strongest risk factor for all-cause, overdose and opioid-related mortality after release was increased age. African Americans, Hispanic persons and Asian persons showed a significant decreased risk in all-cause, overdose and opioid-related death (compared with white persons). Increased length of incarceration was associated with a small decreased risk in all-cause and overdose death. |
| Binswanger 2016 et al. [23] | Multivariable model for overdose mortality after release: substance dependence (adjusted OR 2.33; 95% CI 1.32-4.11), history of injection drug use (adjusted OR 2.43; 95% CI 1.53-3.86), panic disorder (adjusted OR 3.87; 95% CI 1.62-9.21), psychiatric prescriptions before release (adjusted OR 2.44; 95% CI 1.55-3.85) and problems with opiates/sedatives in 6 months before incarceration (adjusted OR 2.81; 95% CI 1.40-5.63). Substance use disorder treatment during the index incarceration (adjusted OR 0.57; 95% CI 0.35-0.90) and having a child (adjusted OR 0.56; 95% CI 0.37-0.85) were protective. Hispanic ethnicity (adjusted OR 0.40; 95% CI 0.19-0.87) and other race/ethnicity (adjusted OR 0.16; 95% CI 0.04-0.70) were associated with a reduced risk of overdose death compared with non-Hispanic white. |
| Binswanger 2016 et al. [24] | Australia cohort: 54 (3%) had an underlying infectious disease–related cause and 174 (11%) had a contributing infectious disease–related cause. Of the 54 deaths with an underlying infectious disease–related cause, substance use was a contributing cause in 14 deaths.  U.S cohort: 171 (7%) had an underlying infectious disease–related cause and 254 (10%) had a contributing infectious disease–related cause. Of the 171 deaths with an underlying infectious disease–related cause, substance use was a contributing cause in 49 deaths. |
| Binswanger 2020 et al. [25] | 57.7% of overdose deaths occurred in the community when not on probation/parole, 28.8% occurred on probation, and 12.8% occurred on parole. There were 11 overdose deaths during the first month after release from prison (25.6% of deaths in the first month) [overdose mortality rate of 259.0 per 100,000 person-years; 95%CI 143.4-467.6]. The overdose mortality rates were 194.0 per 100,000 person-years (95%CI 114.9-326.7) and 119.2 per 100,000 person-years (95%CI 29.8-476.6) during the first month following when probation or parole ended, respectively. |
| Bird 2015 et al. [26] | For all deaths in the 12 weeks after prison release, the proportion that were DRDs was 70% (95% CI 66–74) in the younger age group; and 32% (95% CI 27–37) in the group aged 35 years or older. Periods (combined age groups): Before prison-based OST (1996–2002): 305 DRDs occurred in the 12 weeks post-release (80,200 qualifying releases), 3.8 per 1000 releases (95%CI 3.4–4.2). After the introduction of prison-based OST (2003–2007): 154 DRDs occurred in the 12 weeks post-release (70,317 qualifying releases), 2.2 per 1000 releases (95%CI 1.8–2.5).  No change in the proportion occurring in the first 14 days (DRDs or opioid-related DRDs). |
| Bird 2016 et al. [27] | In 2006–2010, 9.8% of opioid-related deaths (ORDs) (193/1970) followed prison release compared to 6.3% of ORDs in 2011–2013 (76/1212, P<0.001; difference of 3.5% (95%CI 1.6–5.4). This translates to 42 fewer prison release ORDs (95% CI 19–65) during 2011–2013. |
| Brinkley-Rubinstein 2018 et al. [28] | Among those with past year incarceration, there were 44 fatal overdoses in 2015 and 35 fatal overdoses in 2014 (total n=79), of which, 35/79 overdose deaths (44%) involved fentanyl. From 2014 to 2015, Fentanyl-involved overdose deaths significantly increased (29% to 56%, p=0.02). The RR for fentanyl-related overdose death among those with past year incarceration in 2015 compared to 2014 was 1.99 (95%CI 1.11–3.57). Among those with past year incarceration, the median number of days from release to death increased from 2014 to 2015 for non-fentanyl-related (99 days to 103 days; p = 0.8344) and only fentanyl-related (40 days to 135 days, p = 0.2735) deaths. |
| Brinkley-Rubinstein 2019 et al. [29] | Of 387,913 incarcerations, 69.3% were positive for substance use disorder (n=268,893) and 33.7% were placed in restrictive housing (n=130,551). 14,086 deaths occurred after release; 1321 (9.4%) were opioid overdose deaths. Those with any time spent in restrictive housing were more likely to have a fatal opioid overdose in the first 2 weeks after release (adjusted HR 2.27; 95% CI 1.16-4.43). There was no dose response for opioid overdose deaths (≥2 restrictive housing placements compared to none) (adjusted HR, 1.24; 95% CI, 0.77-1.98). Those with >14 consecutive days in restrictive housing had no greater risk of opioid overdose mortality within 1 year after release than those with 0 days (adjusted HR, 1.24; 95% CI, 0.80-1.92). The association between restrictive housing and opioid overdose mortality following release among white individuals compared with nonwhite individuals was adjusted HR 1.38 (95%CI 1.03-1.83] and HR 0.82 (95% CI 0.37-1.80). |
| Bukten et al. [30] | Six months post-release overdose mortality rate was 696 per 100,000 person-years. Of all deaths in the first week after release (n=145), 85% were overdose deaths (n=123) with a peak during the 2 days immediately after release. In the second week, 3–4 weeks and 2–6 months, overdose death accounted for 68, 62 and 46% of all deaths, respectively. Compared with the first week, the risk of overdose death was reduced in week 2 (IRR 0.43; 95%CI 0.31–0.59), weeks 3–4 (IRR 0.22; 95% CI 0.16–0.31) and 2–6 months (IRR 0.12; 95% CI 0.10–0.15). Those incarcerated for 3–12 months had a higher risk of overdose death compared with those who were in custody for longer or shorter time periods (IRR 2.79; 95% CI 2.23–3.51). Recidivism was associated with an increased risk of overdose death (former imprisonments IRR 1.31; 95%CI 1.10–1.16). Women had an increased risk of overdose death during the first 6 months after release (IRR 1.42; 95%CI 1.06–1.91). |
| Calcaterra et al. [31] | Among former prisoners, men had a lower risk of cocaine-only related death (HR 0.41; 95% CI 0.22–0.77) and non-cocaine psychostimulant-related deaths and the cocaine only-related deaths combined (HR 0.55; 95% CI 0.32–0.94) compared to women. Increasing age (decade) was associated with an increased risk of cocaine only-related deaths (HR 1.70; 95% CI 1.31–2.21) and cocaine and non-cocaine psychostimulant-related deaths combined (HR 1.59; 95% CI 1.28–1.98). Increased length of incarceration (years) was associated with a decreased risk of non-cocaine psychostimulants deaths (HR 0.42; 95% CI 0.22–0.80) and cocaine-only deaths and non-cocaine psychostimulant deaths combined (HR 0.76; 95% CI 0.63–0.92). The incidence mortality rate (IMR) was highest in the first two weeks after prison release. For cocaine deaths, 14/49 died in the first two weeks after release (IMR 1,224.0; 95% CI 583-1865). Of the non-cocaine psychostimulant-related deaths, 5 of the 25 total deaths occurred in the first two weeks after prison release (IMR 437.1; 95% CI 142–1,020). |
| Chang et al. [32] | The estimated probability of mortality in men, within 5 years following release, was higher with substance use disorders [10·2% (95%CI 9·6–10·7)] than without [3·2% (95%CI 3·0–3·4%)]. In women, the probability of mortality within 5 years after release with substance use disorders was 6·5% (95%CI 5·2–7·8) and without was 2·6% (95%CI 1·8–3·5). The rate of all-cause mortality was increased by substance use disorders (adjusted HR 1·67; 95% CI 1·53–1·83). After release, 925 all-cause deaths in men and 85 in women were potentially attributable to substance use disorders, relating to a PAF of 34% and 50%, respectively. |
| Degenhardt et al. [33] | Accidental drug-induced deaths after release (2000–10)  Total: Males CMR 4.2; 95%CI 3.7-4.7; Females CMR 3.1; 95%CI 2.4-3.9; both CMR 3.9; 95%CI 3.5-4.3 First day: Males CMR 17.0; 95% CI 2.1-61.3; Females CMR 33.5; 95% CI 0.8-186.7; both CMR 20.3; 95% CI 4.2-59.4 First week: Males CMR 25.8; 95% CI 16.0-39.5; Females CMR 24.3; 95% CI 7.9-56.8; both CMR 25.5; 95% CI 16.7-37.4 First 2 weeks: Males CMR 21.9; 95% CI 15.3-30.5; Females CMR 12.4; 95% CI 4.0-28.9; both CMR 20.0; 95% CI 14.3-27.3  First 4 weeks: Males CMR 16.2; 95% CI 12.0-21.4; Females CMR 7.7; 95% CI 2.8-16.8; both CMR 14.5; 95% CI 11.0-18.8 First year: Males CMR 6.9; 95% CI 5.9-8.0; Females CMR 4.9; 95% CI 3.3-6.9; both CMR 6.5; 95% CI 5.6-7.5 |
| Forsyth 2014 et al. [34] | Indigenous former prisoners were less likely to die of drug-related causes than non-Indigenous former prisoners in the first 4 weeks after release (adjusted HR 0.23; 95% CI 0.08–0.64), from >4 weeks up to 6 months after release (adjusted HR 0.27; 95% CI 0.13–0.57) first year after release (adjusted HR 0.34; 95%CI 0.21–0.53) and after more than 1 year after release (adjusted HR 0.35; 95%CI 0.22–0.56).  Indigenous status and cause specific rate ratios of drug-related mortality of different time periods after release from prison  Indigenous grouping Up to 4 weeks RR 4.8, 95% CI 1.6-14.0 >4 weeks to 6 months RR 2.1, 95% CI 0.94-4.9  > 6 months to 1 year RR 3.1, 95% CI 1.4-6.7 > 1 year 1.0 Non-Indigenous grouping Up to 4 weeks RR 7.7, 95% 5.6-10.6 >4 weeks to 6 months RR 3.0, 95% CI 2.4-3.9  > 6 months to 1 year RR 2.0, 95% CI 1.5-2.7 > 1 year 1.0 |
| Forsyth 2018 et al. [35] | Drug-related mortality rate was CMR 3.4 per 1000 person-years; 95% CI 2.0–5.7. SMR 32; 95% CI 19–55. |
| Gan et al. [36] | Overdose related deaths among people with an incarceration history (n=140). Mortality rate 897 per 100 000 person years. Overdose related deaths among people without an incarceration history (n=494). Mortality rate 22 per 100 000 person years. Risk of death drug overdose and incarceration history: unadjusted HR 41.42; 95% CI 34.33–49.98, adjusted HR 4.04; 95% CI 3.23–5.06 |
| Gjersing et al. [37] | Of those released from prison within 6 months before death (n=18), 10 deaths were within 3 weeks after release, of which 8 were within the first 2 weeks. The most common main cause of death was heroin intoxications (83%). Where heroin was not the main cause, 1 death was from morphine intoxications and in 2 deaths no toxicological information was available. |
| Green et al. [38] | Overdose deaths in those recently incarcerated: 2016: (n=26 of 179; 14.5%); 2017: (n=9 of 157; 5.7%). 60.5% reduction in mortality (RR 0.4 95%CI 18.4-80.9%). The number needed to treat to prevent an overdose death was 11 (95%CI 7-43). Overdose deaths in those recently incarcerated, deaths attributed to fentanyl were: 2016 (n=16 of 26; 61.5%); 2017 (n=8 of 9; 88.9%). Overdose deaths in those recently incarcerated, deaths within 30 days of release were: 2016 (n=10; 38.5%); 2017 (n= 1; 11.1%) |
| Groot et al. [39] | SMR (drug intoxication death) in the year after release was 11.59 (95%CI 6.38–16.79). 20% of deaths (n=137) occurred within one week of release and 9% of deaths (n=63) occurred within two days of release. Time after release to death, median 78 days and mean 113 days. Deaths: 56% were associated with mixed substance toxicity (n= 391), 77% were due to opioid toxicity alone or mixed drug toxicity including opioids (n = 538). The most common non-opioid substance causing death was cocaine (n=73, 10%). |
| Haas et al. [40] | There was no significant difference in fatal overdoses between those who received methadone in incarceration or not (unadjusted OR 0.52 95% CI 0.23-1.17). No significant difference in time to fatal overdose for those who continued methadone treatment during incarceration and those who did not (HR 1.94 95%CI 0.86-4.38). Resuming methadone after release was protective against fatal opioid overdoses (OR 0.26 95%CI 0.11-0.62). |
| Hacker et al. [41] | Of the 211, 109 (51.7%) overdosed within 90 days and 54 (25.6%) overdosed within 30 days after their last release. |
| Hakansson et al. [42] | Total deaths n=166. Drug-related causes of death:  Accidental overdose n=44 (27%). Substance-use disorder n=3 (2%). Injury/intoxication with undetermined intent n=20 (12%). Association with death after release: Lifetime history of substance use Heroin HR 1.88; 95%CI 1.27–2.79; Drug overdose HR 1.55; 95%CI 1.08–2.22. |
| Huang et al. [43] | 48 (34%) drug overdose deaths. MMT attendance had a protective effect for overdose mortality (Cox proportional hazard model) (HR=0.09, P = 0.02). 16 (11%) deaths due to drug-related infections, such as endocarditis, septicemia or pneumonia. 13 deaths occurred within the first week after release [mortality rate=13.7/100 person-years] (compared to the next 4 weeks [mortality rate =3.2/100 person-years, relative rate RR 4.3, P < 0.001]. Of the 13 deaths which occurred in the first week, 7 deaths were by drug overdose and 1 death was a drug-related infection. |
| Kinner et al. [44] | Drug-related deaths after release (Western Australia and New South Wales):  WA cohort: 23.3% within 4 weeks of release and 30.9% within 1 year of release. NSW cohort: 51.0% within 4 weeks of release and 44.5% within 1 year of release. |
| Kouyoumdjian et al. [45] | Overdose deaths: 2.3 (95%CI 0.6– 6.0) per 1000 person-years in the first 2 weeks after release and 0.4 (95%CI 0.3–0.5) per 1000 person-years after 26 weeks post-release. |
| Krawczyk et al. [46] | Those released from incarceration during the study period (26.61%) had a higher risk of overdose mortality than those who remained detained for the duration of the study period (adjusted OR 3.02; 95%CI 1.87−4.88). Number of months incarcerated was not associated with overdose risk (OR 0.99; 95%CI 0.97–1.02). |
| Larochelle et al. [47] | Fatal opioid overdose standardized mortality ratios (SMR) and population attributable fractions (PAF) associated with release from incarceration in past 12 months. Person years (%): 14,686 (0.2%) and opioid deaths (%): 126 (9.6%). Opioid death incidence rate: 858 per 100,000 person years; SMR 30.0; 95% CI 24.8-35.3 and PAF 0.09; 95%CI 0.08-0.11.  Fatal opioid overdose SMR and PAF associated with release from incarceration in past 12 months, by age group 11-49 years: Opioid deaths: 113 (11%). Opioid death incidence rate: 901 per 100,000 person years; SMR 30.3; 95% CI 24.7-35.9 and PAF 0.11; 95%CI 0.09-0.13 ≥50 years: Opioid deaths: 13 (4%). Opioid death incidence rate: 606 per 100,000 person years; SMR 27.8; 95% CI 12.7-42.9 and PAF 0.04; 95% CI 0.02-0.06  Fatal opioid overdose SMR and PAF for fatal opioid overdoses associated with release from incarceration in past 12 months, by sex. Female: Opioid Deaths: 39 (10%). Opioid death incidence rate: 1,082 per 100,000 person years; SMR 92.4; 95% CI 63.4-121 and PAF 0.10; 95% CI 0.07-0.13 Male: Opioid Deaths: 87 (9%). Opioid death incidence rate: 785 per 100,000 person years; SMR 23.0; 95% CI 18.2-27.9 and PAF 0.09; 95% CI 0.07-0.11  Fatal opioid overdose standardized mortality ratios and population attributable fractions associated with release from incarceration, by varied exposure windows. 0-3 months: SMR 43.2; 95% CI 32.6-53.8  4-12 & not 0-3 months: SMR 21.0; 95%CI 15.8-26.2 13-24 & not 0-12 months: SMR 16.6; 95%CI 12.3-20.9 25-36 & not 0-24 months: SMR 13.2; 95%CI 8.9-17.6  0-3 months: PAF 0.05; 95%CI 0.04-0.06 0-12 months: PAF 0.09; 95%CI 0.08-0.11 0-24 months: PAF 0.13; 95%CI 0.11-0.15 0-36 months: PAF 0.16; 95%CI 0.14-0.18 |
| Lim et al. [48] | Drug-related death after release: SMR 2.2; 95% CI 1.9-2.5 Drug-related death during the first 2 weeks after release: SMR 8.0; 95% CI 5.2-11.8 Drug-related death during 3-4 weeks after release: SMR 4.2; 95% CI 2.1-7.3  Drug-related death during 5-6 weeks after release: SMR 3.7; 95% CI 1.8-6.8  Drug-related death during 7-8 weeks after release: SMR 2.0; 95% CI 0.6-4.6  Drug-related death during ≥9 weeks after release: SMR 1.9; 95% CI 1.6-2.2  After release, non-Hispanic white had a high risk drug-related death SMR 5.2; 95% CI 4.0-6.6 Highest risk of drug-related deaths was among white women SMR 8.6; 95% CI 3.7-16.9 Higher risk of drug-related death in high-income neighborhoods among people released SMR 3.3; 95% CI 2.2-4.9  Among people released, the drug-related mortality rate during the first 2 weeks was 3.8 times higher than in 5 weeks after release or later (95% CI 1.9, 7.7) Non-Hispanic white race/ethnicity and older age were associated with a high risk of drug-related death. People released from incarceration who lived in homeless shelters had elevated rates of drug-related mortality (RR 3.4; 95% CI 2.1-5.5). For drug-related deaths, more time spent in custody was associated with a reduced predicted mean number of days between release and mortality. |
| Loeliger et al. [49] | Of 170 deaths with reported cause, 26 were drug overdose as the primary reported cause of death (15·3%). In the first year after index or most recent release, among deaths, time-to-death from drug overdose/accidental injury was less than from HIV/AIDS complications. |
| Marsden et al. [50] | First year after release mortality rate:  n=160 deaths: 1.22 per 100 person years.  n=102 drug-related poisoning (DRP) deaths: 0.78 per 100 person years.  Person follow-up time, mortality rates and number of deaths: 1 to 28 days (1,133 person years): DRP 1.58 per 100 person years (18 deaths) 29 to 121 days (3,521 person years); DRP 0.68 per 100 person years (24 deaths) 122 to 365 days (8,478 person years); DRP 0.71 per 100 person years (60 deaths)  In first 4 weeks, n=18 DRP deaths; opioid substitution treatment (OST) exposed group n=3 (mortality rate was 0.47 per 100 person years) and OST unexposed group n=15 (mortality rate was 3.06 per 100 person years); unadjusted HR 0.15; 95% CI 0.04-0.53. After 4 weeks, the difference narrowed and there was no evidence of between-group difference in DRP mortality risk apparent during the 2nd-4th month or 5th month to 1 year. For DRP deaths, there was no evidence of mediation between community treatment and OST exposure (ratio of HR 1.26; 95% CI 0.07-21.29); Likelihood Ratio Test (LRT) P value 0.86).  6,140 (40.6%) were admitted to drug misuse treatment in the first 4 weeks after release. For DRP deaths risk, there was no statistical association with community drug misuse treatment (HR 0.39; 95% CI 0.11-1.36) |
| Pizzicato et al. [51] | N=837 overdoses deaths; there were 146 overdose deaths in the first four weeks and 107 overdose deaths in the first two weeks after release. The overdose mortality rate was 257 deaths per 100,000 person-years (95%CI 240–275). The most common drug class involved in overdose deaths was opioids (detected in 83.5% (n=699) of overdose deaths).  Overall overdose death released: SMR 5.29; 95% CI 4.93–5.65 (female SMR: 12.65, 95% CI: 10.87–14.43 and male SMR: 4.50, 95% CI: 4.15–4.84) All race/ethnicity groups had a higher risk of overdose death among released people (highest for white, non-Hispanic individuals SMR 11.23; 95% CI 10.19–12.28) Overdose death among released people: 0-2 weeks after release: SMR 36.91; 95% CI 29.92–43.90, 3-4 weeks after release: SMR 13.86; 95% CI 9.51–18.21 and ≥5 weeks after release: SMR 4.53; 95% CI 4.19–4.87.  Those released with a serious mental illness were at higher risk of overdose death (Multivariate HR 1.54; 95% CI 1.27–1.87). Incarceration between 31 days to 6 months had a higher risk of overdose death (Multivariate HR 1.32; 95% CI 1.13–1.54) compared to incarceration ≤30 days. Incarceration >1 year was Multivariate HR 0.56; 95% CI 0.32–0.97. |
| Ranapurwala et al. [52] | Opioid overdose death (OOD) among former prisoners: 2-weeks after release SMR 40.5; 95% CI 29.7-51.3, 1-year after release SMR 10.6; 95% CI 9.5-11.7 and complete follow-up SMR 8.3; 95% CI 7.8-8.7. Heroin deaths among former prisoners: 2 weeks after release SMR 74.4; 95% CI 42.6-106.3, 1-year after release SMR 17.7; 95% CI 14.6-20.9 and complete follow-up SMR 14.3; 95% CI 12.9-15.7.  Standardized death rates for prescription opioid, methadone, and synthetic narcotic overdose were very high. OOD rate among released increased from year following release in 2000 (54 per 100 000 person-years (95% CI 22-86)) to year following release in 2015 (179 per 100 000 person-years (95% CI 125-233))   At complete follow-up, released individuals who were in receipt of in-prison mental health treatment had increased risk of OOD (adjusted HR 1.9; 95% CI 1.7-2.2). At complete follow-up, in-prison intermediate to longterm substance abuse treatment was associated with a small increase in OOD (adjusted HR 1.2; 95% CI 1.0, 1.4). OOD risk in first 2 weeks after release, aged 26–50 years was adjusted HR 7.0 (95% CI 1.7,28.8) and ≥51 years was adjusted HR 8.7 (95% CI 1.8,42.5), compared to those aged 18-25 years. Increased OOD risk for men in the first 2 weeks (adjusted HR 2.4; 95% CI 0.88, 6.7) (not for complete follow-up). > 2 previous incarcerations had a higher risk of opioid overdose mortality: 2 week OOD risk, 1-2 previous incarcerations adjusted HR 1.3; 95% CI 0.63-2.5 and >2 previous incarcerations adjusted HR 2.1; 95% CI 1.1-4.2.  Incarceration because of drug-related conviction had a higher OOD risk: 2 week OOD risk, adjusted HR 2.0; 95% CI 1.1, 3.7. Race was strongly associated with OOD risk: 2 week OOD risk adjusted HR 6.1; 95% CI 3.1-11.7, 1-Year OOD Risk adjusted HR 8.8; 95% CI 6.5-11.9 and complete Follow-Up OOD Risk adjusted HR 11.3; 95% CI 9.6-13.3. |
| Rosen et al. [53] | Mortality from opioid poisoning (illicit & prescription, underlying primary poisoning and secondary contributing opioid cause of  deaths) released from 2008–2015, by incarceration hospitalization status (N=111,479) All: n=460 deaths. Death rate 97.6 per 100,000 person-years Hospitalisation during incarceration: n=8 deaths. Death rate 198.9 per 100,000 person-years Not Hospitalisation during incarceration: n=452 deaths. Death rate 96.7 per 100,000 person-years |
| Saloner et al. [54] | Odds ratio of opioid overdose death:  Released from prison adjusted OR 4.23; 95% CI 2.10-7.11 Any arrest OR 1.54; 95% CI 0.65-2.77 Any parole/probation OR 2.00; 95% CI1.53-2.71 Any drug misdemeanour only OR 1.07; 95% CI 0.50-2.30 Any drug felony OR 0.37; 95% CI 0.07-1.37 |
| Spaulding 2011 et al. [55] | After release overall mortality SMR 1.54; 95% CI 1.48-1.61 After release cause of death accidental poisoning (drug overdose) SMR 3.48; 95% CI 2.76-4.33 |
| Spaulding 2015 et al. [56] | 2 deaths by accidental poisoning (likely drug overdose) in the 14 days after release (rate ratio of 2.91 relative to later deaths from the same cause (P=.19)). Overdose during the 30 days following release (5/3248 person-years) compared to after 30 days was statistically significance (rate ratio 3.46; P=.02).  Cause of Death: accidental poisoning  Total (n = 3863, Person-Years = 424 524): accidental poisoning n=123 (3%) Inside prison (n = 655, Person-Years = 158 481): accidental poisoning n=1 (0%) ≤14 days after any release (n = 22, Person-Years = 1515): accidental poisoning n=2 (9%) 15–30 days after any release (n = 18, Person-Years = 1733): accidental poisoning n=3 (17%) > 30 days after any release (n = 3133, Person-Years = 262 795): accidental poisoning n=117 (4%) |
| Spittal 2014 et al. [57] | CMR drug-related deaths was 14.6 per 10,000 person-years (95% CI 13.3-16.2). Higher drug-related death rate in the first 2 weeks after any release (CMR 114.0 per 10,000 person years, 95% CI 70.9-183.4), than during the following 24 weeks (CMR 27.2 per 10,000 person years, 95% CI 20.6-35.7). Drug-related deaths, CMR was 33.9 deaths per 10,000 person-years (95% CI 26.6-43.1) during the first six months after any release, and 13.2 per 10,000 person-years (95% CI 11.8-14.7) after this. |
| Spittal 2019 et al. [58] | Drug overdose after release model Use of heroin or other opioids in the community: adjusted OR 4.85; 95% CI 1.99–11.79 Two or more custodial sentences: adjusted OR 2.93; 95% CI 1.20–7.14 Married at beginning of current sentence: adjusted OR 0.58; 95% CI 0.25–1.34. Antidepressants during current sentence: adjusted OR 2.15; 95%CI 0.65–7.12. Ever made a suicide attempt: adjusted OR 0.98; 95% CI 0.38–2.54. Ever had problematic alcohol use in the community: adjusted OR 1.01; 95% CI 0.44–2.30. |
| Van Dooren et al. [59] | Drug-related death within 365 days of index release, by sex.  Young at index release (<25 years): Male n=35 (43%) Female n=5 (50%)  Older at index release (≥25 years): Male n=73 (30%) Female n=6 (25%)  Relative risk of death 0.9 (95% CI 0.6–1.3)  Opioid-related deaths within 365 days of index release, by sex.  Young at index release (<25 years): Male n=21 (26%) Female n=1 (10%)  Older at index release (≥25 years): Male n=43 (17%) Female n=3 (13%)  Relative risk of death 0.9 (95% CI 0.5–1.4) |
| Victor et al. [60] | 2017: n=85 fatal overdoses. Among those who had not experienced a fatal overdose as of 1 January 2018: 2018 n=73 fatal overdoses (mortality rate 262 per 100,000 people). Within the county in 2018, the overdose mortality rate was 35.74 per 100 000 (95% CI 33.03–37.37). In those released from jail, the rate of overdose was 7.32 (95% CI 6.44–8.21) times higher than that of the surrounding community. 2019: n=79 fatal overdoses.  Overdose mortality increased for each prior booking (adjusted HR 1.21; 95% CI 1.15-1.28). Overdose mortality for the presence of an unlawful possession of a syringe charge at the most recent jail booking was adjusted HR 3.55; 95% CI 2.55-4.93.  Hazard of fatal overdose: Age (in years) HR 1.018; 95% CI 1.01–1.03. Race: black/African American HR 0.488; 95% CI 0.36–0.66. Race: other HR 0.663; 95% CI 0.09–4.75. Sex: female HR 1.113; 95% CI 0.84–1.47. Additional post-release bookings HR 0.970; 95% CI 0.91–1.03. |
| Webb et al. [61] | Relative risk for suicide by specific methods:  Risk of poisoning by narcotics & hallucinogens: male violent offenders: unadjusted OR 18.66 (95% CI 11.37–30.60) and adjusted OR 5.82 (95% CI 3.27–10.38). Male nonviolent offenders; unadjusted OR 3.22 (95% CI 2.23–4.65) and adjusted OR 2.00 (95% CI 1.33–2.99). Risk of poisoning by analgesics, antipyretics & antirheumatics: male violent male offenders adjusted OR 1.31 (95% CI 0.40–4.28) and nonviolent male offenders adjusted OR 2.40 (95% CI 1.33–4.32). Risk of poisoning by psychotropic drugs: male violent offenders: adjusted OR 1.68 (95% CI 0.89–3.16); male nonviolent offenders: adjusted OR 1.39 (95% CI 0.98–1.98).  Fatal self-poisoning with narcotics & hallucinogens in violent women offenders (OR 32.4, CI 13.1-80.2); nonviolent women offenders (OR 5.9, CI 4.0-8.5). |
| Wortzel et al. [62] | Cause of death - overdose: total deaths n=103 (Veteran person-time of risk 7,447; Non-veteran person-time of risk 49,602) Veteran deaths n=21; Veteran death rate 282 per 100,000 person-years Nonveteran deaths n=82; Nonveteran death rate 165 per 100,000 person-years Veteran status and risk of overdose death: adjusted HR 1.36; 95%CI 0.81-2.26 |

**Appendix 9**

**Appendix 9 Forest plots for standardized mortality ratios (SMRs) across the included studies, grouped by time periods examined after release**

**Any time after release**

**First 2 weeks after release**

**First 3-4 weeks after release**

**First year after release**

**Appendix 10 Summary of variables investigated in included studies**

Included studies used hazard ratios to investigate the association between drug-related deaths/overdose mortality and socio-demographic (sex/gender, age, race/ethnicity and veteran status), custody (increasing years of incarceration, length of incarceration, number of previous incarcerations, incarceration because of drug-related conviction, restrictive housing, time period after release, each prior booking and unlawful possession of a syringe charge at the most recent jail booking), substance-use and treatment (lifetime history of substance use heroin, continued methadone treatment during incarceration, MMT attendance, opioid substitution treatment exposure and in-prison intermediate to long-term substance abuse treatment) and mental health (serious mental illness and receipt of in-prison mental health treatment). Included studies used odds ratios to investigate the association between drug-related death/overdose mortality and demographics (race/ethnicity, married at beginning of current sentence and having a child), custody (violent offenders, number of months incarcerated and two or more custodial sentences), substance-use and treatment (substance dependence, history of injection drug use, use of heroin and other opioids in the community, ever had problematic alcohol use in the community, problems with opiates/sedatives in 6 months before incarceration, substance use disorder treatment during the index incarceration and resuming methadone after release) and mental health (antidepressants during current sentence, psychiatric prescriptions before release, panic disorder and ever made a suicide attempt).

Other methods for reporting deaths after release from prison included proportions of deaths from drug-related causes (e.g. as a percentage) and drug-related deaths within specific time periods. Proportions were often reported by age (in years) at death and type of drug involved in overdose death. Probability of death within time period and population attributable fractions (PAF) were reported. Mortality rates were often reported including incidence mortality rate, overdose or drug-related poisoning mortality rates and mortality rate by hospitalisations during incarceration. Rates between time periods after release were reported, as was incidence rate ratios (IRR) comparing risk of overdose death in weeks after release (association between overdose and recidivism and sex/gender also reported by IRR). Rates of drug-related deaths/overdose mortality were investigated for years, age, sex/gender and use of homeless shelters.

Some studies reported drug-related deaths in relation to a change in policy or introduction of programme or treatment. One study investigated the risk of drug-related deaths and proportion of deaths within the first two weeks after release, both before and after the introduction of a prison-based opioid substitution therapy policy [26]. A separate study investigated any association before and after the introduction of Scotland’s National Naloxone Programme (NNP) and opioid-related death after prison release within 4 weeks [27]. Marsden reported drug-related poisoning deaths after release among a prison-based opioid substitution treatment exposed group and non-exposed groups [50].
